# Alternative polyadenylation dependent function of splicing factor SRSF3 contributes to cellular senescence

**DOI:** 10.18632/aging.101836

**Published:** 2019-03-04

**Authors:** Ting Shen, Huan Li, Yifang Song, Li Li, Jinzhong Lin, Gang Wei, Ting Ni

**Affiliations:** 1 State Key Laboratory of Genetic Engineering and Ministry of Education (MOE) Key Laboratory of Contemporary Anthropology, Collaborative Innovation Center of Genetics and Development, Human Phenome Institute, School of Life Sciences and Huashan Hospital, Fudan University, Shanghai 200438, China; 2 State Key Laboratory of Genetic Engineering, School of Life Sciences, Fudan University, Shanghai 200438, China

**Keywords:** senescence, alternative polyadenylation, *SRSF3*, *PTEN*, 3′UTR

## Abstract

Down-regulated splicing factor SRSF3 is known to promote cellular senescence, an important biological process in preventing cancer and contributing to individual aging, via its alternative splicing dependent function in human cells. Here we discovered alternative polyadenylation (APA) dependent function of SRSF3 as a novel mechanism explaining SRSF3 downregulation induced cellular senescence. Knockdown of *SRSF3* resulted in preference usage of proximal poly(A) sites and thus global shortening of 3′ untranslated regions (3′ UTRs) of mRNAs. *SRSF3*-depletion also induced senescence-related phenotypes in both human and mouse cells. These 3′ UTR shortened genes were enriched in senescence-associated pathways. Shortened 3′ UTRs tended to produce more proteins than the longer ones. Simulating the effects of 3′ UTR shortening by overexpression of three candidate genes (*PTEN, PIAS1* and *DNMT3A*) all led to senescence-associated phenotypes. Mechanistically, SRSF3 has higher binding density near proximal poly(A) site than distal one in 3′ UTR shortened genes. Further, upregulation of *PTEN* by either ectopic overexpression or *SRSF3*-knockdown induction both led to reduced phosphorylation of AKT and ultimately senescence-associated phenotypes. We revealed for the first time that reduced SRSF3 expression could promote cellular senescence through its APA-dependent function, largely extending our mechanistic understanding in splicing factor regulated cellular senescence.

## Introduction

Alternative splicing plays an important role in cellular senescence and aging [1–5]. Core splicing machinery and related splicing factors undergo dramatic changes during aging [6], accompanied with global splicing changes of downstream target genes [7–9]. Heterogeneous nuclear ribonucleoproteins (hnRNPs) and serine/arginine-rich (SR) splicing factors (SRSFs) are two groups of factors regulating alternative splicing and play important roles in numerous biological processes including aging [5,10–12]. Known examples include that the expression changes of multiple such factors (*Hnrnpa1*, *Hnrnpa2b1*, *Sf3b1*, *Srsf3, etc.*) are associated with mice lifespan and some (*HNRNPA1* and *HNRNPA2B1*) are even related to parental longevity in humans [13]. Notably, decreased expression of splicing factor SRSF3 (also known as SRp20) is found in multiple cellular senescence models, and depletion of SRSF3 intriguingly induces cellular senescence via its influence on the choice of *TP53* splicing isoforms in human fibroblast cells [5]. Reversely, elevated SRSF3 expression level is universal in many cancers, which can promote cell growth and maintain the transformation properties of cancer cells [14]. It has also been reported that higher expression of SRSF3 and the consequent splicing dysfunction is associated with neurodegenerative diseases and cancers [15–17]. These findings suggest that splicing-dependent function of SRSF3 plays an important role in senescence and related biological processes.

However, increasing evidence has come to highlight the biomedical importance of revealing splicing-independent function of splicing factors in fully understanding their regulation mechanism [18,19]. As an example, splicing factor RBFox2 directly interacts with Polycomb complex 2 (PRC2) to regulate genome-wide transcription in mammals [19]. In addition, RBFox2 binding to 3′ UTR of *Jph2* gene can antagonise miR34a-mediated gene suppression and plays a role in heart failure [20]. Splicing factor SRSF3 can directly bind to transcripts of histone *H2a* gene to facilitate their nucleus-to-cytoplasm transport [21]. Interestingly, SRSF3 can modulate the translation efficiency of a viral RNA through interacting with an RNA-binding protein PCBP2 [22]. Noteworthy, SRSF3 can also regulate the alternative poly(A) (pA) site recognition in calcitonin coding gene *CALCA* by affecting CSTF2 binding [23]. These above findings on splicing-independent function of SRSF3 inspire us to hypothesize that alternative polyadenylation (APA) dependent function of SRSF3 could also play a role in regulating cellular senescence.

APA is a phenomenon that one gene contains multiple polyadenylation (pA) sites to produce transcript isoforms differ either at the lengths of 3′ untranslated regions (UTR-APA) or C-terminal domains (CR-APA) [24,25]. UTR-APA is more prevalent than CR-APA at genome-wide level [25], which could lead to distinct difference in RNA stability, translation efficiency, localization of RNA and protein among isoforms with different lengths of 3′ UTR [26,27]. The dynamic APA changes have been reported to occur in multiple physiological or pathological processes [28–32]. Global 3′ UTR shortening due to the favorite usage of the proximal pA site took place in cell proliferation and tumorigenesis, and genome-wide lengthening of 3′ UTRs occurs during development and differentiation [33]. It has been discovered that APA regulation is widespread in eukaryotes, and there are more than 70% genes in human genome undergoing APA [25,34], further supporting the prevalence and importance of APA. As for the regulation mechanisms, the *cis*-acting elements and 3′ end processing factors can both affect pA site selection [24,33,35–37]. For example, CSTF2 is a well-known factor that participates in mRNA 3′ end processing, and its cellular concentration can affect pA site usage [38,39]. Knockdown of *CSTF2* plus its paralog *CSTF2t* can promote genes to preferentially use the distal pA site [40,41]. Besides, CFIm25 and CFIm68 were another two 3′ end processing factors that have been reported to be involved in pA site selection. Favorite usage of the proximal pA site was observed when *CFIm25* or *CFIm68* was down-regulated [42–45]. Polyadenylation can also be coupled with splicing [46], recent studies demonstrated that multiple splicing factors (such as U1 snRNP [47,48], HnRNP H/H’ [49] and NOVA2 [50]) could regulate APA. Additionally, factors of other aspects, such as transcription [51], chromatin state [52] and other RNA binding proteins [53–55], can also be involved in the modulation of APA.

To examine whether down-regulation of splicing factor SRSF3 promotes cellular senescence via its APA-dependent mechanism, we performed transcriptome-wide APA profiling on *SRSF3*-knockdown (*SRSF3*-KD) and control cells by PA-seq [56] (a 3′ end specific enrichment RNA-seq method) and strand-specific RNA-seq methods [57]. Interestingly, we observed *SRSF3*-KD induced global shortening of 3′ UTRs in both human and mouse cells. SRSF3 has higher binding density near proximal pA sites than distal ones in 3′ UTR shortened genes. These 3′ UTR-shortened genes were enriched in senescence-associated pathways, and shortened 3′ UTRs tended to produce more corresponding proteins. We further found that mimicking the effect of 3′ UTR shortening by overexpression of three candidate genes promoted senescence-associated phenotypes. These results combined to support the model that APA-dependent function of SRSF3 depletion can lead cellular senescence.

## RESULTS

### Down-regulation of SRSF3 leads to global shortening of 3′ UTR in human and mouse cells

**Figure 1:**
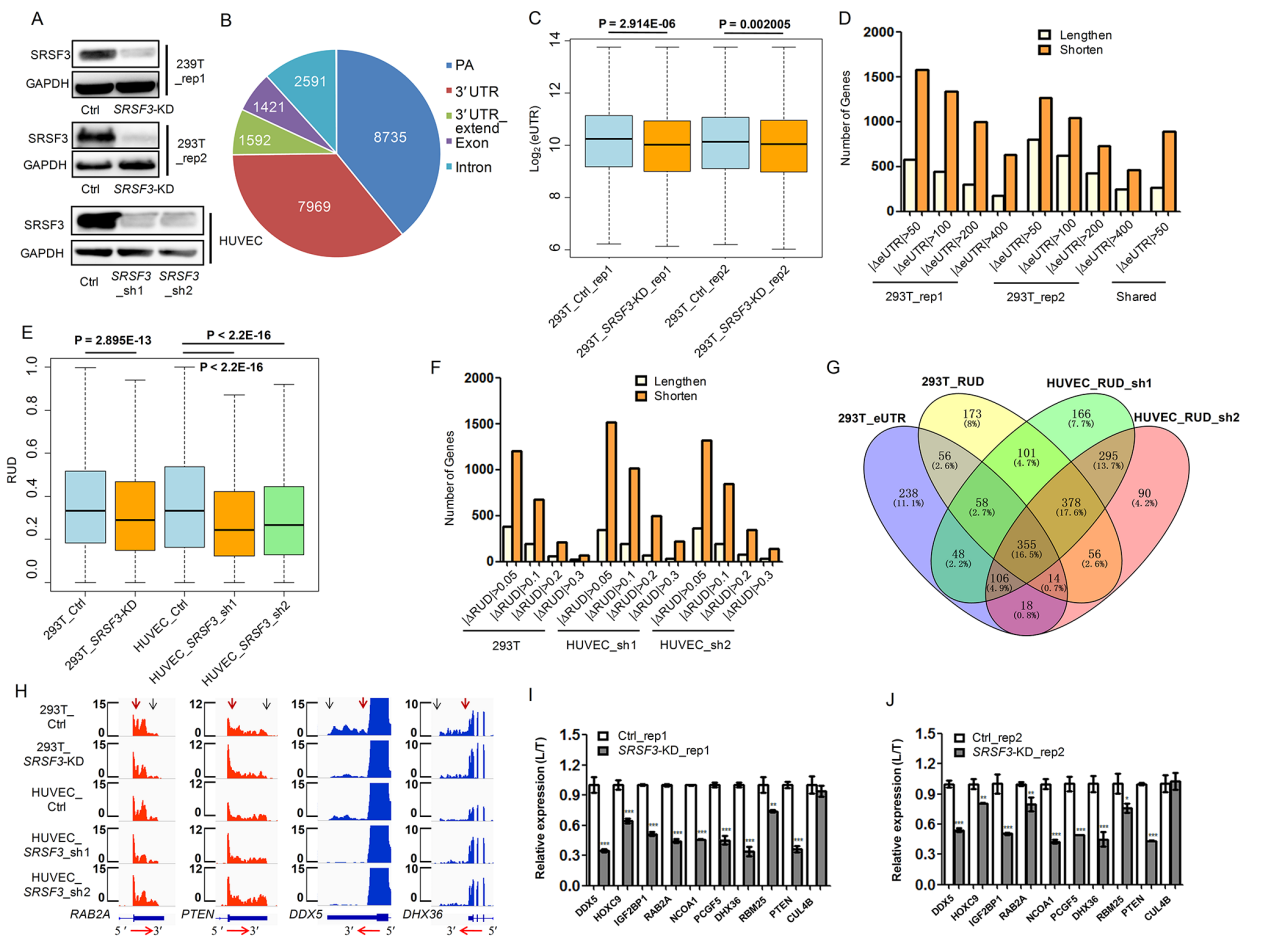
**SRSF3 downregulation leads to global shortening of 3′ UTR in human cells.** (**A**) Western blot confirmed lentivirus-mediated RNA interference in both human 293T and HUVEC cells. GAPDH served as internal loading control. (**B**) Genomic distribution of pA sites identified in 293T cells based on PA-seq method. (**C**) Box plot of log_2_-transformed eUTR based on PA-seq in control and *SRSF3*-KD 293T cells. The P value of *t*-test is shown. (**D**) Histogram of gene numbers with 3′ UTR shortening or lengthening upon *SRSF3* KD at different cutoffs and overlapped genes with shortened 3′ UTR (with the cutoff of |ΔeUTR| > 50) between two biological replicates in 293T cells. |ΔeUTR| > 50, 100, 200 and 400 represent the absolute difference of eUTR between *SRSF3*-KD and control 293T cells, respectively. Number of shared genes (labelled Shared) between two biological replicates were also shown. (**E**) Box plot of RUD in 293T and HUVEC cells upon knockdown of *SRSF3*. (**F**) Histogram of gene numbers with 3′ UTR shortening or lengthening upon SRSF3 KD at different ΔRUD cutoffs. |ΔRUD| > 0.05, 0.1, 0.2 and 0.3 each represents a threshold of absolute difference of RUD between *SRSF3*-KD and control human cells. (**G**) Venn diagram of genes with shortened 3′ UTR based on different methods (eUTR and RUD), different shRNAs (sh1 and sh2) and different cells (293T and HUVEV) (ΔRUD ≤ -0.05) upon knockdown of *SRSF3*. (**H**) RNA-seq tracks of four representative genes in two human cell types upon *SRSF3* KD. The transcription direction is shown at the bottom. The vertical red and blue arrows represent the proximal and distal pA sites, respectively. Y axis denotes the normalized read coverage. (**I, J**) qRT-PCR validation of the usage of longer 3′ UTR in the total expression (L/T) in both control and *SRSF3*-KD 293T cells of two biological replicates (rep1 in I and rep2 in J). Rep1 and rep2 represent two biological replicates, and sh1 and sh2 denote two different shRNAs. ** and *** mean P value less than 0.01 and 0.001 (*t*-test), respectively.

To examine whether *SRSF3-*KD induces downstream changes other than alternative splicing, we firstly applied our published PA-seq protocol, which specifically enriched 3′ ends of mRNA by reverse transcription with modified oligo(dT) primer to capture the polyA tail and precisely identify polyadenylation site at the genome scale [56], to detect global APA changes in human 293T cells. Two biological replicates of lentivirus-mediated short hairpin RNA (shRNA) interference were performed and down-regulation of SRSF3 protein was confirmed by western blot (Fig. 1A, Fig. S1). The reliability of the identified pA sites was analyzed before comparing the dynamic changes of APA between *SRSF3*-KD and control cells. Known pA sites and those located at 3′ UTR regions were the top two categories of the identified pA sites (Fig. 1B), consistent with previous reports [32,56]. Besides, 85.5% of the identified pA sites were covered by PolyA_DB3 [58] and nucleotides composition near pA sites were in line with previous reports (Fig. S2). Moreover, canonical polyA signals (AAUAAA and AUUAAA) occupy ~75% of identified pA sites (Fig. S2C). These quality control results demonstrated the satisfied quality of the identified pA sites and their reliability for further analyses. The changes of pA site usage upon *SRSF3* knockdown were next analyzed. Effective 3′ UTR (eUTR), which considering both location and abundance of pA sites for genes with APA, was used to reflect the weighted length of 3′ UTR for each gene [32,56]. Interestingly, an overall 3′ UTR shortening pattern evaluated by eUTR was observed in *SRSF3-*KD human 293T cells with two biological replicates (Fig. 1C), suggesting that SRSF3 downregulation favored the usage of proximal pA sites. We further examined the eUTR changes at individual gene level and found that *SRSF3*-KD induced more genes to use proximal pA sites in both replicates (Fig. 1D). Notably, a considerable proportion of overlapped genes between two biological replicates using PA-seq method and eUTR calculation further supported the reproducibility of such global trend (Fig. 1D).

As an independent validation, we next adopted the method of RUD index [59], which reflected the relative usage of distal pA sites compared to total pA sites, to confirm the APA changes based on a separate RNA-seq data. Consistent with the results based on eUTR method, we detected a global reduction of RUD index upon *SRSF3* KD in 293T cells (Fig. 1E), suggesting the favoring of proximal pA sites and shortening of 3′ UTRs. At individual gene level, we also detected more genes using shortened 3′ UTRs than lengthened ones in *SRSF3*-KD 293T cells (Fig. 1F). To expand this conclusion in more human cells, we applied the same RNA-seq and RUD analysis in Human Umbilical Vein Endothelial Cells (HUVECs), which is widely used as a vascular senescence model [32,60–62]. In line with the results in human 293T cells, knockdown *SRSF3* with two replicates in HUVECs both displayed a similar 3′ UTR shortening trend at both genome-wide (Fig. 1E) and individual gene level (Fig. 1F).

To gain a comprehensive comparison of genes tending to use shorter 3′ UTRs upon *SRSF3*-KD based on different methods, biological replicates and types of cells, the interrelation of these gene sets was shown in a venn diagram (Fig. 1G). The majority (1134 genes) of *SRSF3*-KD induced 3′ UTR shortened genes were shared between two biological replicates of HUVECs (Fig. 1G). A considerable overlap (483 genes) between two different bioinformatical methods (eUTR and RUD) was also detected in 293T cells (Fig. 1G). Importantly, there were 355 genes showed 3′ UTR shortening in both 293T and HUVECs based on different methods and biological replicates (Fig. 1G), which were probably the common targets of SRSF3 in different cell types. *SRSF3*-KD induced 3′ UTR shortening in four representative genes was visualized in tracks of RNA-seq (Fig. 1H) and PA-seq (Fig. S3). Ten candidate genes were further selected for validation by reverse transcription coupled with quantitative real-time polymerase chain reaction (qRT-PCR), nine of which were confirmed to have reduced usage of distal pA sites (*i.e.*, favor the proximal pA sites) in *SRSF3-*KD human cells (Fig. 1I-J). These above results indicated that downregulation of *SRSF3* caused global 3′ UTR shortening in human cells.

To examine whether *Srsf3* could play a similar APA regulatory role in mouse cells, we knocked down *Srsf3* in mouse embryonic fibroblasts (MEFs) (Fig. S4A) and constructed RNA-seq libraries followed by RUD analysis. Consistent with the trend in human cells, knockdown of *Srsf3* in MEFs also led to global shortening of 3′ UTRs (Fig. S4B) and the majority of genes with APA changes favored proximal pA sites (Fig. S4C). Both visualization of RNA-seq results and qRT-PCR validation of selected genes supported the shortening of 3′ UTR in *Srsf3-*KD mouse cells (Fig. S4D-F). Altogether, our results proved that downregulation of splicing factor SRSF3 resulted in global shortening of 3′ UTR in both human and mouse cells.

### SRSF3 favors proximal pA sites binding and transcriptionally modulates APA

**Figure 2:**
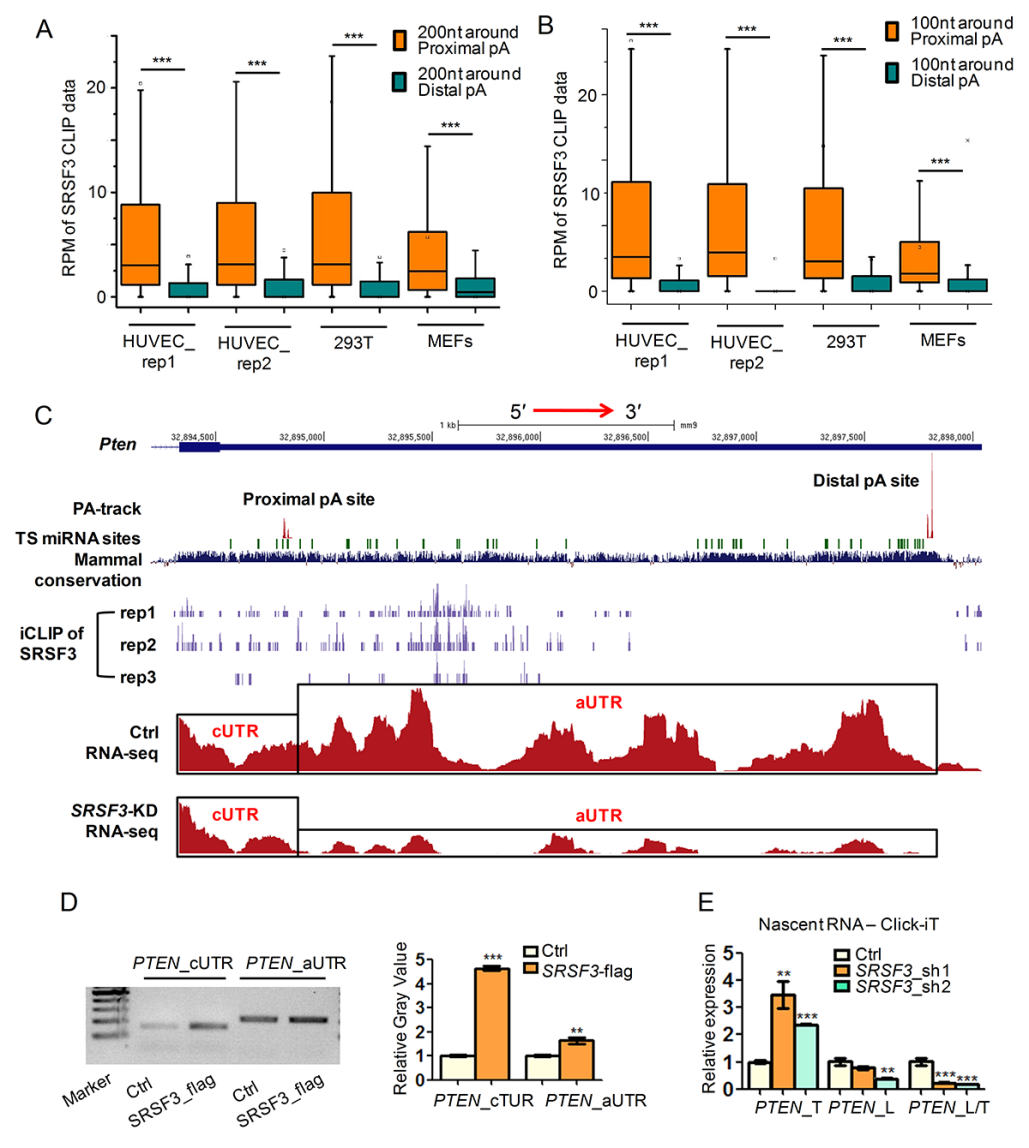
**SRSF3 favors binding proximal pA sites and modulates APA at transcriptional level.** (**A-B**) Box plots of public available human and mouse CLIP-seq data of SRSF3 in 3′ UTR shortened genes. Y axis represents the normalized tag intensity (reflected by RPM, reads per million) within 200 nt (**A**) or 100 nt (**B**) around the proximal and distal pA sites. Rep1 and rep2 means two biological replicates of HUVEC cells. (**C**) A combinated UCSC genome view near the 3′ UTR of mouse *Pten* containing PA-seq track, TargetScan predicted microRNA binding sties (TS miRNA sites), Mammal conservation score, SRSF3 iCLIP track (three replicates), RNA-seq track of control (Ctrl) and *SRSF3*-KD cells . cUTR and aUTR represent the common and alternative 3′ UTR of *Pten*, respectively. (**D**) Semi-quantitative RIP-PCR using cUTR or aUTR specific primer for *PTEN* on immunoprecipitated products separately harvested by anti-flag (SRSF3_flag) and IgG (Ctrl). IgG served as background binding (Ctrl). Left panel denotes the gel image while right panel represents the relative gray value quantified by imageJ software. (**E**) qRT-PCR quantifying the nascent RNA isolated by using the Click-iT kit in *SRSF3*-KD (sh1 and sh2) and control (Ctrl) 293T cells. *PTEN*_T, *PTEN*_L and *PTEN*_L/T represent total expression (short plus long 3′ UTR), expression of long 3′ UTR and relative expression ratio of long 3′ UTR compared to total expression, respectively. *, ** and *** mean P value less than 0.05, 0.01 and 0.001 (*t*-test), respectively.

We next examined whether SRSF3 directly regulated alternative polyadenylation by integrative analysis of public SRSF3 CLIP-seq (crosslinking-immunoprecipitation and high-throughput sequencing) data [63,64] and our PA-seq and RNA-seq data before and after *SRSF3* knockdown. As we focused on UTR-APA, CLIP signal located at 3′ UTRs was analyzed. Interestingly, the binding intensity of SRSF3 was significantly higher near proximal pA sites than distal ones for 3′ UTR shortened genes in both human and mouse cells (Fig. 2A). Since about 90% of genes had ≥ 100 nucleotides (nt) distance between proximal pA site and stop codon (Fig. S5), we analyzed CLIP signal within 100 nt around proximal or distal pA sites and observed same results (Fig. 2B). These results suggested that SRSF3 globally favors the proximal pA site binding. To confirm such result at individual gene level, three candidate genes (PTEN, a well-known tumor suppressor and related to longevity; DNMT3A, a known methyltransferase associated with aging and cancer; PIAS1, a repressor of transcription factor STAT1 that related to breast tumorigenesis), which were validated undergoing 3′ UTR shortening in *SRSF3*-KD cells (Fig. 1I-J, Fig. S4E-F), were visualized with their CLIP-seq and RNA-seq signal in UCSC genome browser. Noteworthy, SRSF3 had higher binding signal around the proximal pA site than the distal one for *PTEN* in all three biological replicates in mouse cells (see iCLIP of SRSF3 track in Fig. 2C, Fig. S6). SRSF3 iCLIP tracks of *PIAS1* and *DNMT3A* showed similar proximal pA site preference (SFig. S7, S8). Consistent with the shortening of 3′ UTR, RNA-seq tracks of these three candidate genes showed considerable ratio change between alternative 3′ UTR (aUTR) and constitutive 3′ UTR (cUTR) in *SRSF3*-KD samples (Fig. 2C, Fig. S7, S8). Importantly, the 3′ UTRs of *PTEN, PIAS1 and DNMT3A* are all evolutionarily conserved and ranked at top 14%, 13.6% and 10.6% in all human coding genes, respectively (Fig. S9), implying the importance of such regulatory role of SRSF3 in an evolutionary view.

Alternative polyadenylation contributing to different isoforms of *PTEN* has been reported by other research [65–67], however, SRSF3 directly binding to its 3′ UTR and regulating its APA is novel, we performed further experimental validations in human cell. RNA immunoprecipitation coupled with semi-quantitative PCR (RIP-PCR) showed higher binding signal of SRSF3 near the proximal pA site than the distal one of *PTEN* (Fig. 2D). These results indicated that SRSF3 regulated APA of *PTEN* by its binding preference to the proximal pA site. To further explore whether SRSF3 regulated *PTEN*’s APA at the transcriptional level, we applied analysis on nascent RNA. qRT-PCR showed that *SRSF3-*KD increased the usage of proximal pA site in nascent poly(A)+ RNA, indicating SRSF3 regulated APA of *PTEN* at transcriptional level (Fig. 2E). Together, SRSF3 favored proximal pA site binding of *PTEN* and regulated its APA at transcriptional level.

### *SRSF3-*KD induced 3**′** UTR-shortened genes enrich in senescence-associated pathways

**Figure 3:**
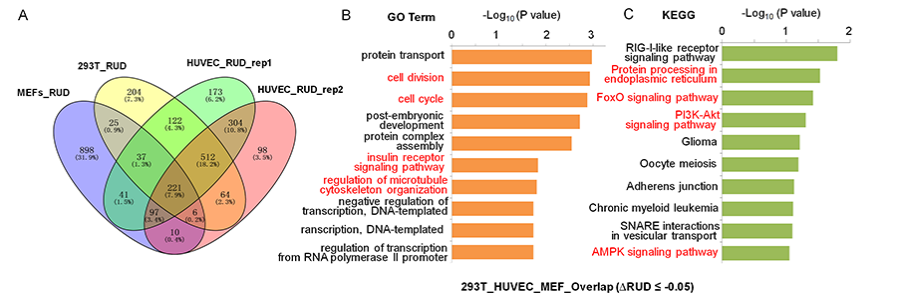
**GO and KEGG enrichment analyses for 3′ UTR shortened genes shared by MEFs, 293T and HUVEC cells when knocking down *SRSF3*.** (**A**) Venn diagram of 3′ UTR shortened genes (RUD-based) upon *SRSF3*-KD in different cells. Both the numbers and percentages were indicated. (**B-C**) GO term (**B**) and KEGG pathway (**C**) enrichment analysis for genes with 3′ UTR shortening shared by *SRSF3*-KD MEFs, 293T and HUVEC cells (221 genes in panel A). Red fonts represent functional categories related to senescence or aging.

To understand the functional consequence of *SRSF3-*KD induced 3′ UTR shortening, functional enrichment analyses were performed on those 3′ UTR shortened genes using gene ontology (GO) and KEGG pathway (Fig. 3A). We first analyzed 3′ UTR-shortened genes shared by two biological replicates of HUVEC, and discovered that four (cell division, protein ubiquitination, cell cycle and Wnt signaling pathway) out of the top ten enriched GO terms were associated with senescence (Fig. S10A) [68,69]. And seven out of the top ten enriched KEGG pathways (Endocytosis, Protein processing in endoplasmic reticulum, Ubiquitin mediated proteolysis, Insulin signaling pathway, mTOR signaling pathway, AMPK singling pathway and FoxO signaling pathway) were associated with senescence or aging (Fig. S10B) [70–73]. Next, we analyzed *SRSF3*-KD induced 3′ UTR shortening genes shared by 293T and HUVEC cells, and found that they were enriched in senescence-associated GO terms (cell division, cellular response to DNA damage stimulus, cell cycle and protein ubiquitination) and senescence/aging related pathways (Protein processing in endoplasmic reticulum, Ubiquitin mediated proteolysis, Endocytosis, FoxO signaling pathway and mTOR signaling pathway) (Fig. S11) [68,70,71,74]. 3′ UTR-shortened genes in MEFs were also enriched in senescence/aging related pathways (cell division, Ras signaling pathway, Wnt signaling pathway, Ubiquitin mediated proteolysis, PI3K-Akt signaling pathway and Endocytosis) (Fig. S12). Finally, we analyzed *SRSF3*-KD induced 3′ UTR shortening genes shared by 293T, HUVEC and MEF cells (221 genes showed in Fig. 3A), and the result showed that they were enriched in senescence-associated GO terms (cell division, cell cycle, insulin receptor signaling pathway and regulation of microtubule cytoskeleton organization) and senescence/aging related pathways (Protein processing in endoplasmic reticulum, FoxO signaling pathway, PI3K-Akt signaling pathway and AMPK signaling pathway) (Fig. 3B,C) [75–77]. These results above indicated that *SRSF3*-KD induced 3′ UTR-shortened genes possibly had the potential to function in senescence and aging in both human and mouse cells.

### Decreased SRSF3 causes senescence-related phenotypes in human and mouse cells

**Figure 4:**
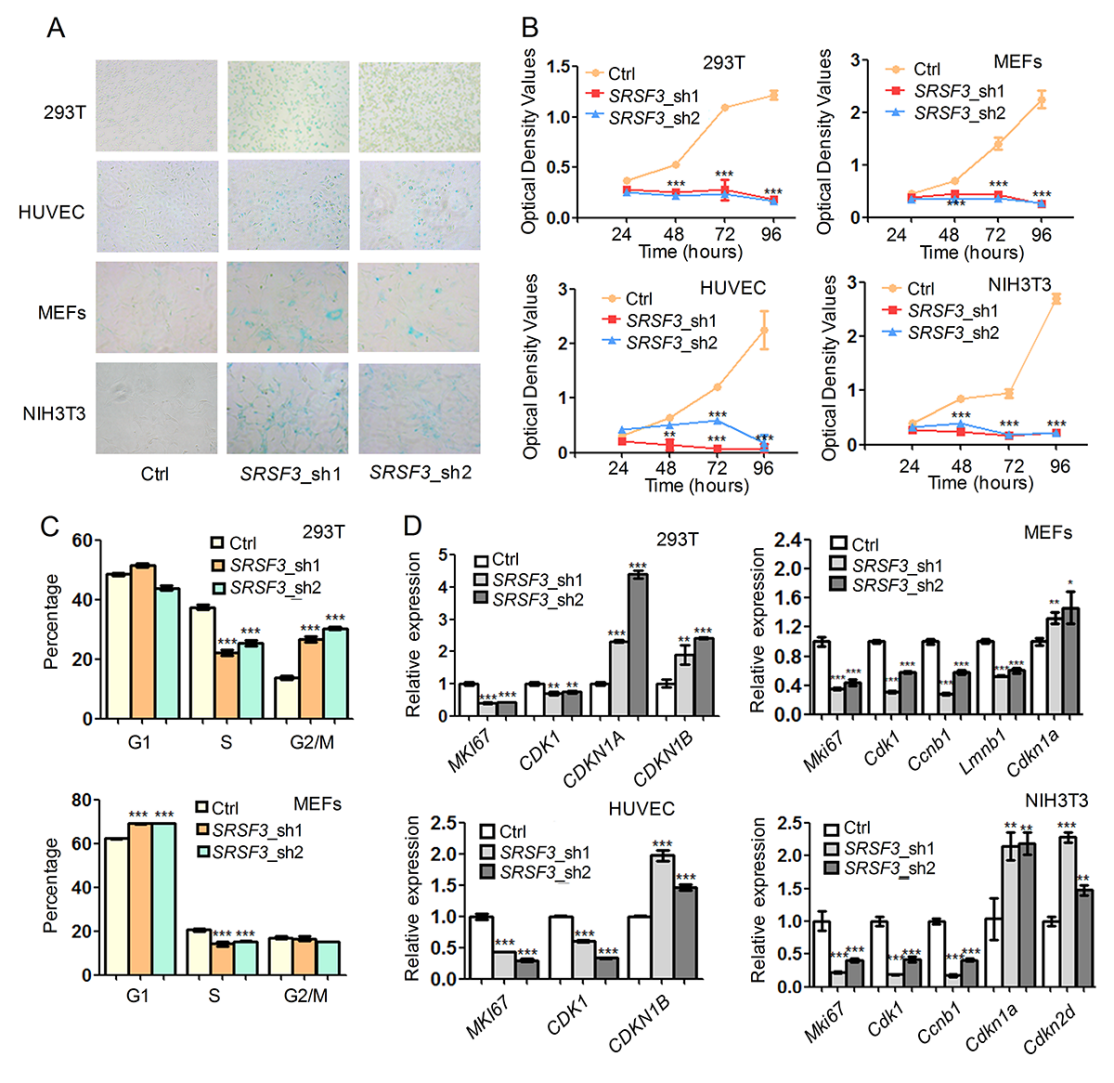
**SRSF3 downregulation leads to senescence-associated phenotypes in both human and mouse cells.** (**A**) SA-β-Gal staining before and after knockdown with two shRNAs (sh1 and sh2). (**B**) CCK-8 assay for cells with or without knockdown of *SRSF3* in human and mouse cells. (**C**) Cell cycle analysis before and after knockdown of *SRSF3* in 293T and MEF cells. (**D**) qRT-PCR revealed that knockdown of *SRSF3* led to decreased expression of cell proliferation markers (*MKI67, CDK1*) and increased expression of senescence marker (*CDKN1A* or *CDKN1B*) in both human (293T and HUVEC) and mouse (MEFs and NIH3T3) cells. *, ** and *** mean P value less than 0.05, 0.01 and 0.001 (*t*-test), respectively.

We next examined whether knockdown of *SRSF3* could lead to senescence-associated phenotypes in human and mouse cells. RNA interferences using two shRNAs targeting *SRSF3* caused increased senescence-associated β-galactosidase (SA-β-gal) staining [78] in both human (293T and HUVEC) and mouse (MEF and NIH3T3) cells (Fig. 4A). In addition, *SRSF3-*KD reduced cell growth rate in tested cell lines (Fig. 4B). Further investigation showed that *SRSF3-*KD resulted in cell cycle arrest in G2/M phase in 293T cells while arrest in G1 phase in MEFs (Fig. 4C). Notably, *SRSF3-*KD caused a common decrease of S phase percentage in both 293T and MEF cells (Fig. 4C). What’s more, knockdown of *SRSF3* in human and mouse cells also led to the decreased expression of *MKI67*, a molecular marker for cell proliferation (Fig. 4D) [79]. In addition, *SRSF3*-KD resulted in upregulation of senescence-related marker *CDKN1A* (encodes p21) and/or *CDKN1B* (encodes p27) in human and mouse cells (Fig. 4D). These results demonstrated that knockdown of *SRSF3* could induce senescence-related phenotypes in both human and mouse cells.

### ***SRSF3-*KD induced 3**′ **UTR shortening genes promote senescence-related phenotypes via their increased protein levels**

**Figure 5:**
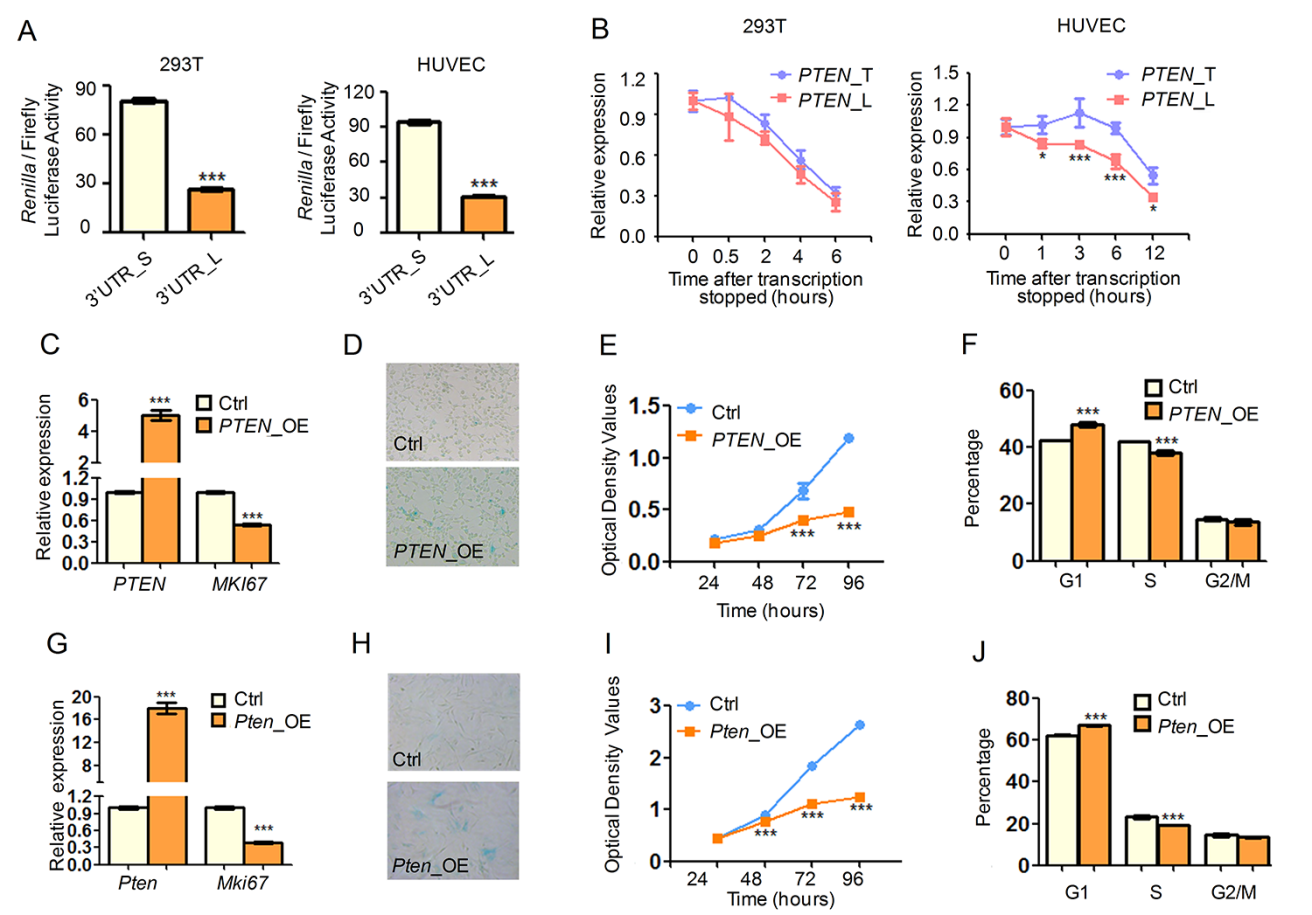
***SRSF3*-KD induced 3′ UTR shortening of *PTEN* produces more protein and contributes to senescence-associated phenotypes.** (**A**) Dual luciferase assay for shorter (S) and longer (L) 3′ UTRs of *PTEN* in 293T (left) and HUVEC (right) cells. (**B**) RNA degradation curve of actD-inhibited transcription cells detected by qRT-PCR using primers specific for longer 3′ UTR (L) or targeting common region shared by longer and shorter 3′ UTR (T) of *PTEN*. (**C, G**) qRT-PCR indicated overexpression of *PTEN* (*PTEN*_OE and *Pten*_OE) led to decreased expression of cell proliferation marker *MKI67* in 293T (**C**) and MEF (**G**) cells. (**D, H**) SA-β-Gal staining for 293T (**D**) and MEF (**H**) cells when overexpressing *PTEN*. (**E, F, I, J**) CCK-8 assay (**E, I**) and cell cycle analysis (**F, J**) for cells with or without overexpression (OE) of *PTEN* in 293T (**E, F**) and MEF (**I, J**) cells. *** and * mean P value less than 0.001 and 0.05, respectively (*t*-test).

As knockdown of *SRSF3* led to global shorting of 3′ UTRs and senescence-associated phenotypes, we hypothesized that 3′ UTR shortening mediated expression change of target genes can be an alternative mechanism in explaining *SRSF3-*KD induced senescence. Our results already showed that SRSF3 directly regulated APA of candidate genes including *PTEN*, *PIAS1* and *DNMT3A* (Fig. 2, Fig. S7, S8). These three genes belong to enriched pathways associated with senescence/aging (*PTEN* belongs to pathways in cancer, PI3K-Akt signaling pathway; *PIAS1* and *DNMAT3A* belong to transcription, DNA-templated, regulation of transcription). We thus chose these genes to test the hypothesis. To examine whether *SRSF3*-KD induced 3′ UTR shortening affects gene expression, we carried out dual luciferase assay with different lengths of 3′ UTRs. It has been reported that protein abundance of the variable *PTEN* isoforms resulting from alternative polyadenylation are distinct because of miRNA effects or difference in protein translation efficiency [65,66,80,81]. Our result showed that transcripts with the shorter 3′ UTR of *PTEN* can markedly produce more protein than those with the longer one in two different human cell types (Fig. 5A, Fig. S13). The result remained true in mouse cells (Fig. S14). In addition, shorter 3′ UTR of *PIAS1* also generated more protein than the longer one (Fig. S15). Interestingly, multiple TargetScan (TS) predicted microRNA (miRNA) binding sites existed in the regions between proximal and distal pA sites for both *PTEN* and *PIAS1* (see TS miRNA sites in Fig. 2C, Fig. S7). This result suggested that shortened 3′ UTR could enhance the protein expression through escaping from targeting by miRNAs. Further, there were more predicted miRNA binding sites within the alternative 3′ UTR of *DNMT3A* (see TS miRNA sites in Fig. S8), however, the dual luciferase assay cannot be performed due to the technical failure of cloning the longer 3′ UTR of *DNMT3A*. Next, RNA turnover rate analysis showed that transcripts with shorter 3′ UTR of *PTEN* was more stable than those with the longer one in HUVEC cells, though a less difference of such stability in 293T cells (Fig. 5B). These results suggested that *SRSF3*-KD induced shortening was likely to increase the protein production of affected genes, possibly contributed by miRNA-mediated stability control, translation efficiency or other mechanisms.

We next mimicked the elevated expression of these 3′ UTR-shortened genes by overexpressing candidate genes and examined whether they could contribute to cell senescence. Overexpression of *PTEN* in human cells led to decreased expression of *MKI67*, a well-known cell proliferation marker (Fig. 5C). Upregulation of *PTEN* also caused senescence-related phenotypes in human cells including increased SA-β-gal activity (Fig. 5D), decreased cell growth rate (Fig. 5E) and reduced percentage of S phase cells (Fig. 5F). Additionally, cells transfected with *PTEN* shorter isoform grew slower than those with the longer one (Fig. S16). Importantly, upregulated *Pten* promoted alike senescence-associated phenotypes in mouse cells (Fig. 5G-J). Besides, we also proved that overexpression of *Pias1* and *Dnmt3a* resulted in similar senescence-related phenotypes, including increased SA-β-gal activity, reduced cell proliferation rate and changed cell cycle (Fig. S17). All these results supported the notion that 3′ UTR shortening contributed to, at least in part, *SRSF3-*KD induced senescence.

PTEN is a well-known tumor suppressor and its overexpression extends mice lifespan through reduced PI3K activity and downstream cancer protection mechanisms [82], consistent with our observation that overexpression of *PTEN* induced cellular senescence (Fig. 5), an important tumor prevention mechanism [83]. Since PTEN can negatively regulate PI3K/AKT pathway through dephosphorylating phosphatidylinositol-3,4,5-trisphosphate (PIP_3_) and thus reduce phosphorylated AKT (p-AKT) abundance [84,85], we next examined whether *PTEN*-induced senescence related to altered level of p-AKT. In good consistence with known reports, overexpressing *PTEN* in 293T and HUVEC cells reduced p-AKT abundance significantly while the AKT level did not exhibit a significant change (Fig. S18A). As knockdown of *SRSF3* induced 3′ UTR shortening of *PTEN* and transcripts with shortened 3′ UTR of *PTEN* generated more protein than those with the longer one (validated by dual luciferase assay, Fig. 5A), one would expect that *SRSF3*-KD can increase the protein level of PTEN. Consistently, *SRSF3* knockdown with two shRNAs both led to higher PTEN protein abundance in human 293T and HUVEC cells (Fig. S18B). Furthermore, *SRSF3* knockdown attenuated the abundance of p-AKT but not that of the total AKT (Fig. S18B), coinciding with the result of PTEN upregulation. Together, *SRSF3-*KD induced senescence can be partially explained by *PTEN* upregulation, which at least in part contributed by 3′ UTR shortening.

## DISCUSSION

**Figure 6:**
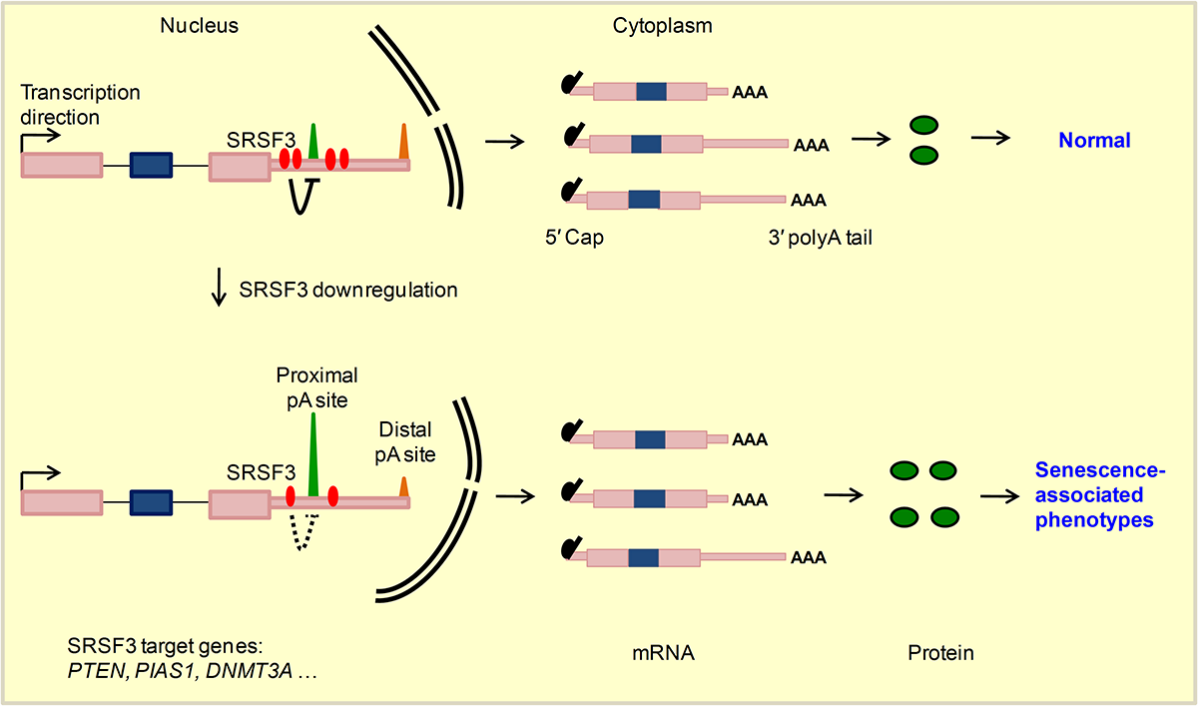
**A working model for SRSF3-mediated 3′ UTR shortening contributes to cellular senescence.** SRSF3 prefers proximal pA sites binding and represses nearby pA sites usage in target genes such as *PTEN, PIAS1* and *DNMT3A* in normal conditions. Upon *SRSF3* knockdown, the repression effect reduced, which in turn leads to the higher usage of corresponding pA sites and 3′ UTR shortening of target genes. Transcripts with shortened 3′ UTR generate more protein, possibly by escaping the miRNA targeting, and finally lead to senescence-associated phenotypes.

Cellular senescence is a cancer prevention mechanism, and SRSF3 could be one of the regulators given SRSF3 is downregulated in multiple senescence models and upregulated in many cancer types [5]. What’s more, knockdown of *SRSF3* induced cellular senescence while increased *SRSF3* expression promoted cancer-related cellular phenotypes further highlighted its regulatory importance in both biological systems [5,14], wherein splicing-dependent function of SRSF3 was mainly focused. However, SRSF3 can also regulate RNA export, RNA stability, alternative polyadenylation and translation [21–23]. Growing evidences have highlighted the biomedical importance of understanding the splicing-independent functions of multiple splicing factors such as RBFox2, SRSF2 and U2AF1 [20,86]. Here, our results also showed that reduction of *SRSF3* expression indeed affected splicing of multiple genes, including *TP53* gene whose splicing pattern change was consistent with the previous research (Fig. S19) [5]. However, splicing-independent function of SRSF3 in cellular senescence has not been explored. In this study, we were surprised to find that knockdown of *SRSF3* led to over one thousand genes favoring proximal pA site usage, resulting in the global shortening of 3′ UTRs in both human and mouse cells. 3′ UTR shortened genes were enriched in senescence-associated pathways and likely produced more protein, as demonstrated by candidate genes. Mimicking the effect of 3′ UTR shortening by overexpression of three candidate genes all caused senescence-related phenotypes. Specifically, SRSF3 regulated *PTEN*’s APA at transcriptional level and contributed to senescence. Thus, *SRSF3-*KD induced senescence can be explained, at least in part, by its APA-dependent function (Fig. 6).

The functional link between *SRSF3-*KD induced APA changes and cellular senescence was supported by multiple evidences. First, *SRSF3-*KD induced 3′ UTR shortened genes were enriched in senescence-associated pathways. Second, overexpression of three candidate genes (*PTEN, PIAS1* and *DNMT3A*), emulating their effect of 3′ UTR shortening upon *SRSF3* knockdown (Fig. 1,2, Fig. S4,S7,S8), promoted senescence-associated phenotypes. Specifically, PTEN, a well-known tumor suppressor and lifespan regulator [82], can be regulated at APA level by SRSF3 in both human and mouse cells. Supporting this, it was recently reported that nuclear poly(A) polymerases also regulated alternative polyadenylation of *PTEN* [66]. We further verified that *SRSF3-*KD induced *PTEN*’s 3′ UTR shortening generated more proteins, leading to reduced level of p-AKT and ultimately senescence-related phenotypes. Additionally, we did not find any obvious changes on the splicing pattern of *PTEN* compared *SRSF3*-KD to the control (Fig. S20). These data combined support the notion that APA-dependent function of SRSF3 contributes to cellular senescence.

To profile the global APA changes in *SRSF3*-KD comparing to the control, we exploited two high throughput sequencing strategies (PA-seq and strand-specific RNA-seq methods) in 293T cells and HUVEC. Although we identified lots of genes undergoing 3′ UTR shortening either by eUTR or RUD analysis on PA-seq and RNA-seq, respectively, the overlap was relative small as showed in Fig. 1G. Three major differences between these two methods may underlie the phenomenon. First, the library construction step is considerable different. eUTR is calculated based on PA-seq data, which specifically enriches 3′ end sequence of polyA+ RNA. However, RUD is calculated based on RNA-seq data, which covers the full length of polyA+ RNA. Second, eUTR considers all identified pA sites located in 3′ UTR while RUD only calculates two pA sites (the most proximal pA site and the most distal pA site) within 3′ UTR. Thus eUTR and RUD will show less overlap considering this issue. Third, eUTR uses tag number in the pA cluster to reflect the usage preference and RUD reflects the relative read coverage on alternative 3′ UTR comparing to common region of 3′ UTR. Lastly, different PCR bias in different genomic region due to factors such as GC context, secondary structure may also underlie some of the difference between eUTR and RUD. Together, these above three major difference may explain the relative small overlap between eUTR and RUD. Consistent with this, overlap between two biological replicates with the same RUD calculation (52.7% for HUVEC_RUD_sh1 and HUVEC_RUD_sh2) is much higher than overlap between eUTR and RUD (22.5%).

Increasing evidences support the idea that splicing factors can regulate alternative polyadenylation. A well-known example was that downregulation of U1 small nuclear ribonucleoproteins (snRNP), which plays an important role in alternative splicing, led to the production of the truncated transcripts resulting from using cryptic pA sites located in introns [47,48]. To regulate alternative polyadenylation located within 3′ UTR, SRSF3 may need to bind to nearby locations. We thus analyzed publicly available SRSF3 CLIP-seq data to confirm whether SRSF3 can bind to 3′ UTR-shortened genes and what’s the binding difference between proximal and distal pA sites. The results showed higher SRSF3 binding density near proximal pA sites than the distal ones (Fig. 2). Given the higher usage of proximal pA site upon *SRSF3* knockdown, the higher SRSF3 binding nearby proximal pA sites possibly inhibited the selection of corresponding pA sites. Further investigation is definitely warranted to elucidate the detailed mechanism of such regulation.

3′ UTR shortening induced protein upregulation probably requires the expression of miRNAs targeting the alternative 3′ UTRs between proximal and distal pA sites. We thus examined whether such miRNAs were expressed by analyzing public small RNA sequencing data in human (293T, HUVEC) and mouse cells (MEFs). Interestingly, the result showed that miRNAs specifically targeting alternative 3′ UTRs of shortened genes have similar expression profiles compared with all expressed miRNAs (Fig. S21), supporting the notion that 3′ UTR shortening produced more protein through escaping of expressed miRNAs [87]. Which miRNA(s) involved in SRSF3-induced cellular senescence deserves extensive experimental validation.

Our recent publication indicated that hundreds of genes underwent 3′ UTR lengthening in mouse embryonic fibroblasts (MEFs) replicative senescence model and rat Vascular Smooth Muscle Cells (rVSMCs) derived from old animal [32]. Specifically, longer 3′ UTR of *Rras2* could produce less protein and induce senescence-related phenotypes [32]. However, it did not rule out the possibility that 3′ UTR shortening of certain genes could also promote senescence, given the complicated and sometimes opposite functions of genes in the same regulatory network. For example, kinase PI3K transforms PIP_2_ to PIP_3_, while phosphatase PTEN reverses PIP_3_ to PIP_2_. The two proteins act oppositely in the PI3K/AKT pathway, and thus similar 3′ UTR length change of these two genes might result in opposite consequences [88,89]. In the present study, we showed that *SRSF3-*KD induced 3′ UTR shortening of *PTEN* could produce more protein and promote senescence-associated phenotypes. These data support a model that both 3′ UTR lengthening and shortening could regulate cellular senescence, and the function of the corresponding gene in the senescence pathway is the key. Keeping in mind that replicative senescence is a complicated biological process that multiple factors including SRSF3 are changed. Although reduced *SRSF3* led to 3′ UTR shortening of target genes, changes of other regulators during replicative senescence may cause 3′ UTR lengthening of target genes. Actually, previous reports demonstrated that both 3′ UTR lengthening and shortening can be observed upon knockdown of different RNA binding proteins [53,54]. Thus, *SRSF3-*KD induced global 3′ UTR shortening is not a contradiction compared to 3′ UTR lengthening in replicative senescence given the changed expression of multiple APA regulators. Understanding the downstream consequence of each APA regulators will definitely extend our understanding of the much-complicated APA changes during cellular senescence.

## MATERIALS AND METHODS

### Cell culture, transfection and selection of stable cell lines

All cells (293T, HUVEC, NIH3T3 and MEF) used in this study were cultured in DMEM medium supplemented with 10% FBS at 37 °C incubator with 5% CO_2_. For transient transfection, cells were seeded in 6-well plate for 60%-70% confluence one day in advance, then transfected with lipofectamine 2000 (Invitrogen) in the second day. Cells were harvested for RNA and protein extraction 48 hours (hrs) after transfection.

For lentivirus transduction to stably knockdown *SRSF3* or overexpress *PTEN*/*PIAS1*/*DNMT3A* in cells, the expression vector or the control vector plus VSVG and gag/pol encoding plasmids was transfected into the 293T cells, respectively. After culture for 24 and 48 hrs, the virus supernatant was harvested to infect cells. 24 hrs post the second infection, the medium was replaced with fresh DMEM medium supplemented with puromycin (final concentration was 2 μg/ml) to screen for stably transformed cells. After one day selection, the survival cells were trypsinized and split into two parts, one for cell proliferation assay and cell cycle analysis, the rest was cultured for additional two days in the medium with puromycin (2 μg/ml) for RNA and protein extraction.

### Vector construction

For *SRSF3* shRNA plasmid construction, the DNA oligonucleotides were annealed and then cloned into the pLKO.1 plasmid by using EcoRI and AgeI restriction enzyme sites. The short and long 3' UTR of *PTEN*/*PIAS1* were PCR amplified by Q5 high-fidelity DNA polymerase (NEB) and then were individually ligated into the downstream of the *Renilla* luciferase coding gene in the dual luciferase vector psiCHECK-2. Firefly luciferase activity serves as the internal control of *Renilla* luciferase expression. To construct ectopic overexpression vector for *PTEN*/*PIAS1*/*DNMT3A*, the complete coding sequences were PCR amplified from human 293T cDNA or mouse 3T3 cDNA and then inserted into the pcDH_EF1_MCS_T2A_Puro vector.

### qRT-PCR and western blot

Total RNA and protein were extracted with TRIzol reagent (Sigma) according to the manufacturer’s instruction. For cDNA synthesis, 1 μg total RNA was reverse-transcribed into cDNA by FastQuant RT Kit (TIANGEN) and oligo(dT) primer. Then quantitative PCR reaction was performed using 2×SYBR mix (KAPA) and the reaction was run on Bio-Rad CFX manager machine. Sequence information of all used primers was listed in Supplementary Table S1.

For western blot, the primary antibody for detecting SRSF3 (Abcam, cat. no. ab198291), PTEN (CST, cat. no. 9188T), pAKT (CST, cat. no. 4060T), AKT (CST, cat. no. 4691T) and GAPDH (CST, cat. no. 2118S) was 1:1000 diluted and incubated for 2 hrs at room temperature. The second antibody (HRP conjugate, YEASON) was 1:5000 diluted and incubated for 1 hr at room temperature. Blots were washed three times with TBST and visualization was carried out by Tanon Chemiluminescent Imaging System.

### RNA stability assay

The stability for short and long isoforms of *PTEN* was measured by adding actinomycin D (actD) at a final concentration of 5 μg/ml into 293T and HUVEC cells to block transcription. Cells were harvested at different time points respectively. Total RNAs from all time points were then extracted by TRIzol reagent. The degradation rate was evaluated by qRT-PCR. The long isoform PTEN_L was quantified using primer pair specific to long 3′ UTR, while the overall expression of PTEN was quantified using primer pair shared by both short and long 3′ UTRs (PTEN_T).

### Dual-luciferase reporter assay

Cells were transfected with psiCHECK-2 vectors containing either short-3' UTR or long-3' UTR using lipofectamine 2000 in 24-well plate. After transfection for 24 hrs, the firefly and *Renilla* luciferase activities were measured one by one according to the manufacture’s instruction (Promega). The final *Renilla* luciferase activity was normalized to the firefly luciferase signal.

### Cell proliferation assay and cell cycle analysis

After selection by puromycin, the survival (transfected) cells were trypsinized and counted using hemocytometer. Then cells were diluted and seeded into a 96-well plate, with 2000 cells per well and three replicates for each time point (add CCK-8 reagent at 24 hrs, 48 hrs, 72 hrs and 96 hrs according to the manufacturer’s protocol). After each treatment, cells were incubated for two hours at 37 °C, then the optical density (OD) at 450 nm and 600 nm were measured respectively for each well by microplate reader (Tecan i-control).

For cell cycle analysis, cells were trypsinized, then centrifuged at 500g for four minutes (mins). Cell pellets were washed once with 1×PBS and resuspended with PBS containing 0.03% Triton X-100 and 50μg/ml Propidium Iodide (PI), and incubate for 10 mins. The cell cycle assay was then performed on the BD Flow Cytometer.

### Nascent RNA assay

The nascent RNA assay was performed with the Click-iT method (Click-iT Nascent RNA Capture Kit from Thermo). Briefly, a nucleotide analog EU was added into the medium and cells were incubated for 1 hr at 37 °C. Total RNA was then extracted, and the EU-contained RNA was biotinylated. The EU-biotinylated RNA was isolated from the total RNA by Dynabeads MyOne streptavidin T1 (Invitrogen). Finally, the EU-RNA was quantified by qRT-PCR.

### RNA immunoprecipitation coupled with semi-quantitative PCR (RIP-PCR)

Aspirate medium from one 10-cm plate 293T cells of 90% confluence, wherein SRSF3-flag is stably overexpressed. Wash cells with cold PBS once. Then irradiate cells under 200 mJ/cm^2^ in UV linker. After UV cross-link, immediately wash cells one time with cold PBS, then add RIP lysis buffer (50 mM Tris pH 7.4, 250 mM NaCl, 5 mM EDTA, 1% NP40, 0.5 mM DTT, 1× protease inhibitor, 100 U/ml RNasin) to the plates, and incubate on ice for 20 min and collect cells in a new 1.5 ml tube. Then sonicate lysed cells using Biorupter, and spin down at 16000g for 10 min at 4 °C to collect the supernatant. Additionally, pre-incubate anti-flag antibody and Rabbit IgG with protein G beads at 4 °C for 4 hours (hrs) separately before harvest cell lysates. Then add one half supernatant to anti-flag-antibody-protein G beads, and the other half to IgG-protein G. After incubate and rotate at 4 °C for 4 hrs, put the tubes in the magnetic separation rack for 1 min. Aspirate the solution and wash beads three times with cold RIP lysis buffer. Then add 4mg/ml proteinase K solution to the beads and incubate for 20 min at 37 °C. Put the tubes in the magnetic separation rack once again, and then add Urea solution (100 mM Tris pH 7.4, 50 mM NaCl, 10 mM EDTA, 7M urea) and incubate another 20 mins at 37 °C. Finally, RNA was extracted by adding TRIzol reagent, and reversely transcribed into cDNA using FastQuant RT Kit (TIANGEN). Then the semi-quantitative PCR was performed using the primers specifically targeting the shared and long 3’ UTR regions of *PTEN*, respectively. Finally, the RIP-PCR result was detected by electrophoresis on 1% agarose gel.

### PA-seq and RNA-seq library construction

PA-seq libraries for global profiling of 3′ UTR length was constructed according to our previous publication [56]. RNA-seq libraries for expression and RUD analysis were constructed according to the previous reports [57,90]. Both PA-seq and RNA-seq libraries were sequenced using Illumina HiSeq platform.

### Bioinfomatical analysis methods

FastQC software was used for quality control of PA-seq and RNA-seq data (http://www.bioinformatics.bbsrc.ac. uk/projects/fastqc/), nucleotides with sequencing quality smaller than 20 were trimmed off. For PA-seq data, paired-end reads were first subjected to strand correction as previously described [56], and only reads heading with “TTT” were kept for further analysis. All the reads (strand-corrected PA-seq reads and quality-controlled RNA-seq reads) were aligned to the reference genome (mm9 for mouse data and hg38 for human data) using STAR [91]. The peak-calling, pA sites identification and localization were processed following our published methods [32,56]. eUTR methods [56] and RUD methods [59] were used for evaluating the changes in pA site usage before and after *SRSF3* knockdown according to the published methods. For RUD analysis in MEF cells, we combined our previously identified pA sites [32] with the RNA-seq data in the present study.

Cufflinks was used to estimate the mRNA abundance using RNA-seq data (fragments per kilobase of transcript per million mapped reads, FPKM) by following previously published instructions [92]. Cuffdiff, a sub-package of cufflinks, was used to evaluate the gene expression changes.

UCSC genome browser was used for visualization of the PA-seq and RNA-seq data on specific genes. For functional enrichment analysis, the Database for Annotation, Visualization and Integrated Discovery (DAVID) [93] was used for the pathway enrichment analysis and the Gene Ontology (GO) and Kyoto Encyclopedia of Genes and Genomes (KEGG) database was selected.

### Data access

The raw PA-seq and RNA-seq data from this study can be accessed at the NCBI Sequence Read Archive (SRA; https://www.ncbi.nlm.nih.gov/sra/) with the accession number PRJNA523954 (or SRP186820).

## SUPPLEMENTARY MATERIAL

Supplementary Figures

Supplementary Table
