# Supplementary material for: Alternative polyadenylation dependent function of splicing factor SRSF3 contributes to cellular senescence
Source: Aging (Albany NY). 2019 Mar 4;11(5):1356–88. doi: 10.18632/aging.101836 (PMC6428108; doi:10.18632/aging.101836)
Supplement: Supplementary Figures [file aging-11-101836-s001.pdf]

SUPPLEMENTARY MATERIAL

Supplementary Figures

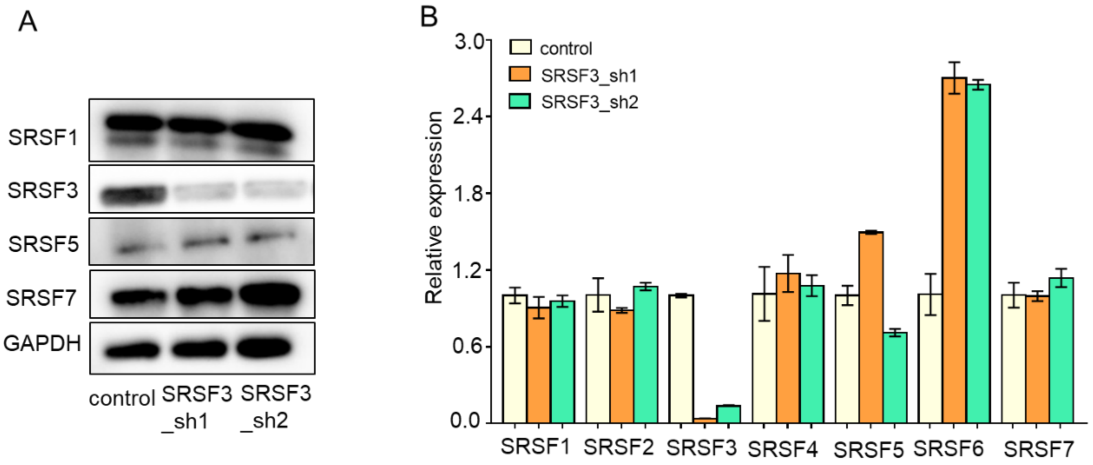

Supplementary Figure S1. *SRSF3*-shRNA specifically reduced SRSF3 protein level. (**A**) Western blot to detect the protein abundance of SRSF1, SRSF3, SRSF5 and SRSF7 before and after shRNA-mediated *SRSF3* knockdown. GAPDH acted as an internal control. (**B**) Relative RNA expression level of SRSF factors, including SRSF1, SRSF2, SRSF3, SRSF4, SRSF5, SRSF6 and SRSF7, in the *SRSF3*-knockdown and control cells evaluated by qPCR.

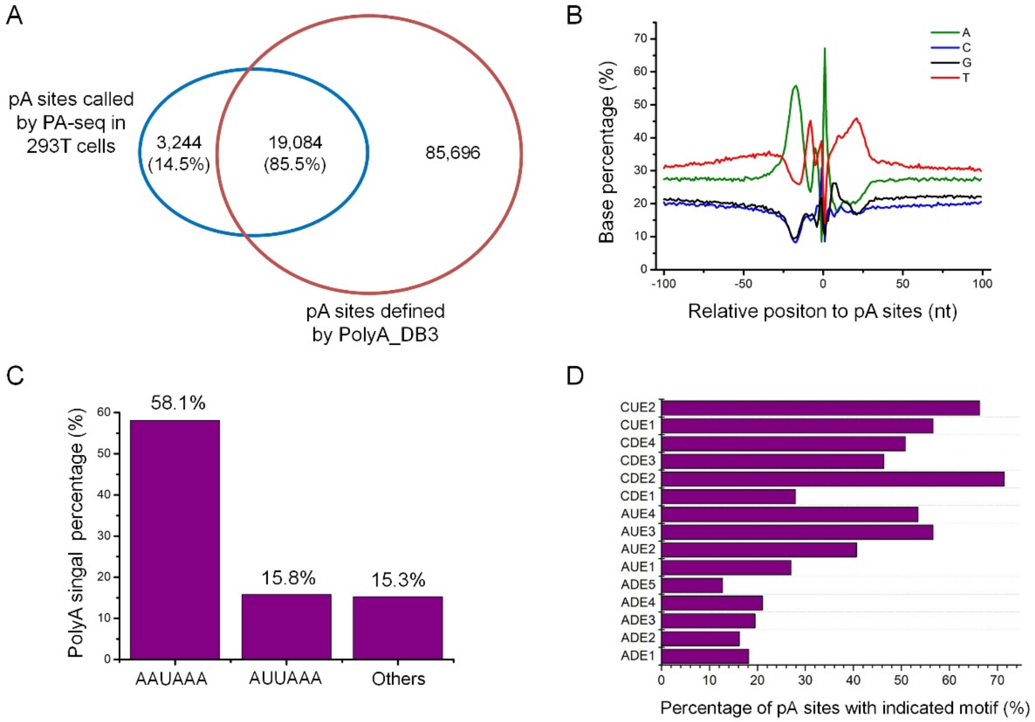

Supplementary Figure S2. Reliability evaluation for identified pA sites using PA-seq in human 293T cells. (**A**) Overlap between identified pA sites and those in PolyA_DB3. (**B**) Base composition analysis of identified pA sites in 293T cells. (**C**) Percentage of two canonical polyA signal (AAUAAA and AUUAAA) and other remaining polyA signals (Others) within 40 nucleotide (nt) upstream the identified pA sites. (**D**) Percentage of polyadenylation motifs predicted by polya_svm [1] in the 200 nt surrounding pA sites.

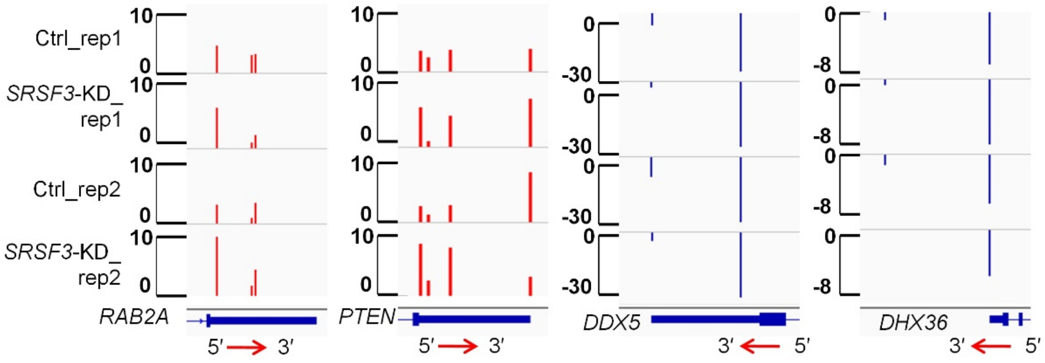

Supplementary Figure S3. PA-seq tracks of genes whose RNA-seq tracks were shown in Fig. 1H. The red arrow indicates the transcription direction. Rep1 and rep2 represent two biological replicates for both control (Ctrl) and SRSF3 knockdown (*SRSF3*-KD) 293T cells. Minus values in the right two panels denote the pA signal belongs to the minus strand.

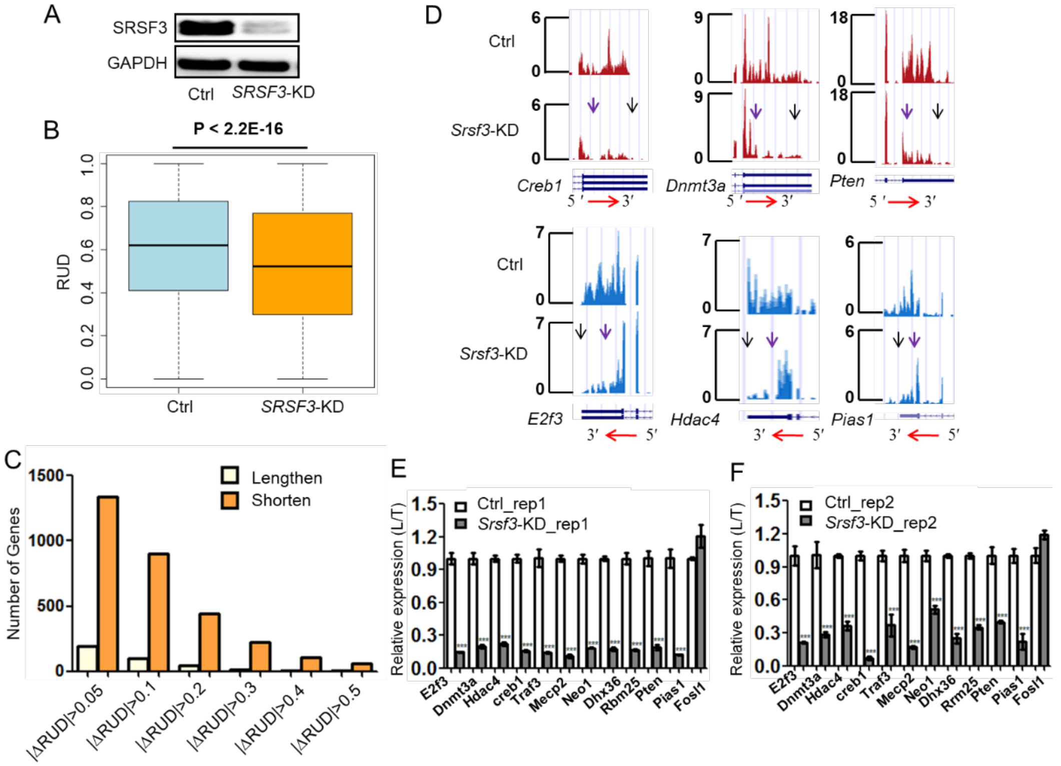

Supplementary Figure S4. SRSF3 downregulation leads to global shortening of 3′ UTR in MEFs. (**A**) Western blot confirmed lentivirus-mediated RNA interference in MEF cells. GAPDH served as internal loading control. (**B**) Box plot of RUD values based on RNA-seq in control (Ctrl) and *SRSF3*-KD MEFs. The P value of *t*-test is shown. (**C**) Histogram of gene numbers with 3′ UTR shortening or lengthening upon SRSF3 KD at different ΔRUD cutoffs. |ΔRUD| > 0.05, 0.1, 0.2, 0.3, 0.4, 0.5 each represent a threshold of absolute difference of RUD between *SRSF3*-KD and control MEFs. (**D**) RNA-seq tracks of six representative genes in MEFs upon *SRSF3* KD. The transcription direction is shown at the bottom with horizontal red arrows. The vertical purple and black arrows represent the proximal and distal pA sites, respectively. Y axis denotes the normalized read coverage. (**E, F**) qRT-PCR validation of the usage of longer 3′ UTR in the total expression (L/T) in both control and *SRSF3*-KD MEFs with two biological replicates (rep1 in E and rep2 in F). *** means P value less than 0.001 (*t*-test).

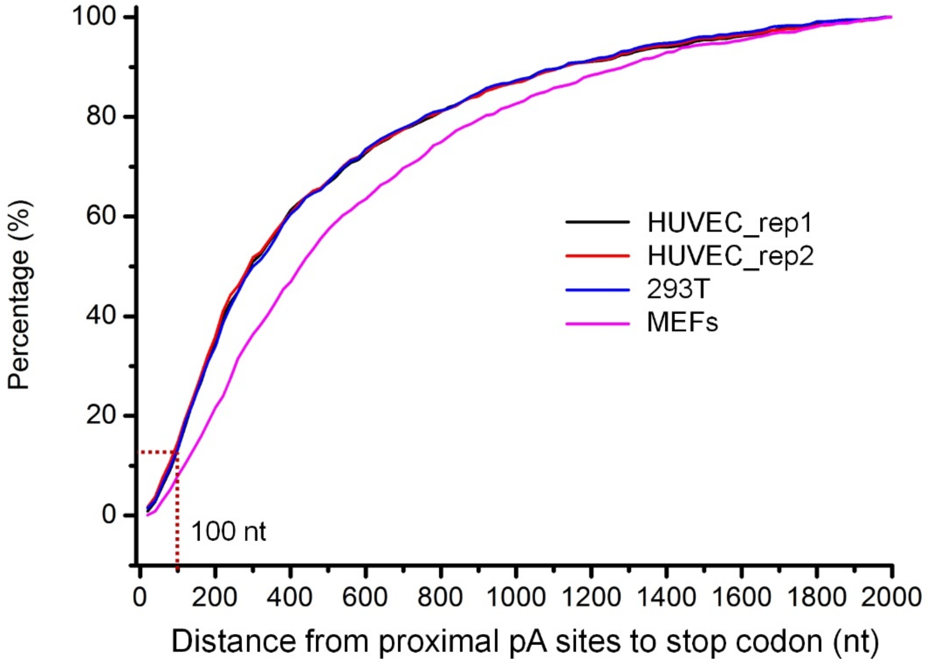

Supplementary Figure S5. Cumulative plot of distance from proximal pA sites to stop codon. The dashed red line indicates ~90% of proximal pA sites have distance more than 100 nt to the stop codon in all indicated cells.

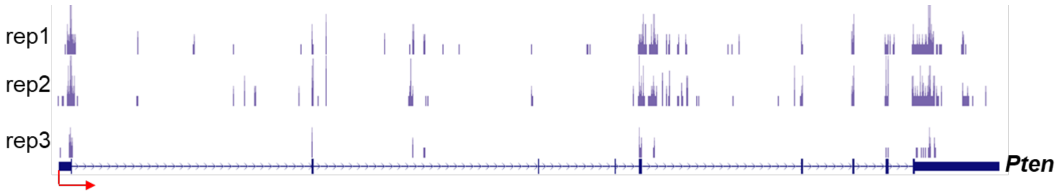

Supplementary Figure S6. iCLIP-seq tracks of SRSF3 on mouse *Pten* gene. Rep1, rep2 and rep3 represent three biological replicates. The red arrow indicates the transcription direction.

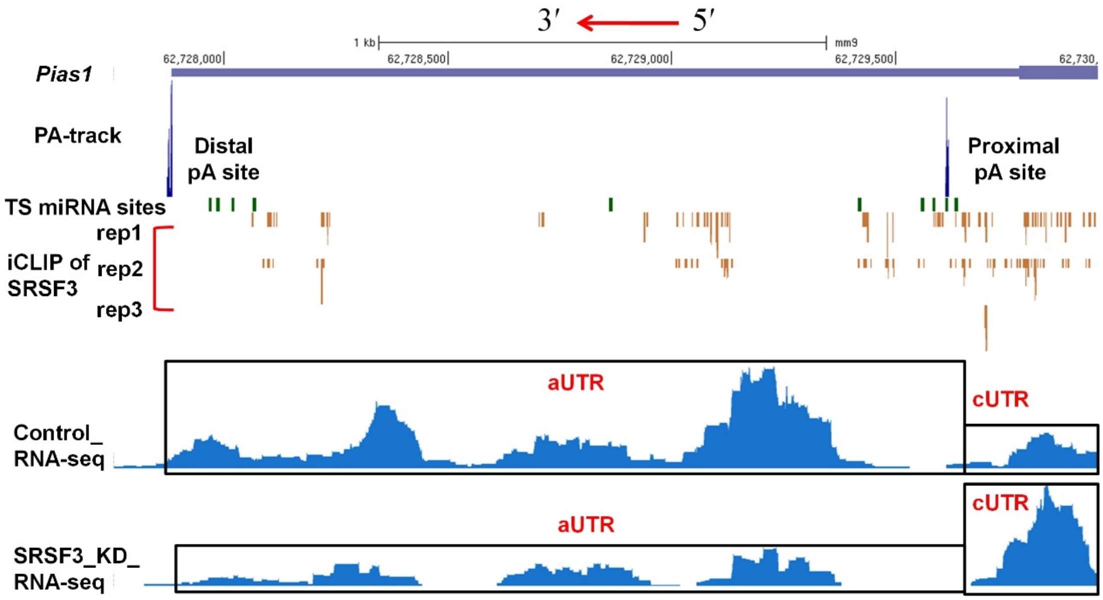

Supplementary Figure S7. SRSF3 preferentially binds around proximal pA site of *Pias1* in mouse cells. A combination of PA-seq track, TargetScan predicted microRNA binding sites (TS miRNA sites), three replicates (rep1, rep2 and rep3) of SRSF3 iCLIP track, RNA-seq track of control (Ctrl) and *SRSF3*-KD cells near the 3′ UTR of mouse *Pias1*. cUTR and aUTR represent common and alternative 3′ UTR of *Pias1*, respectively. Red arrow at the top represents the transcription direction.

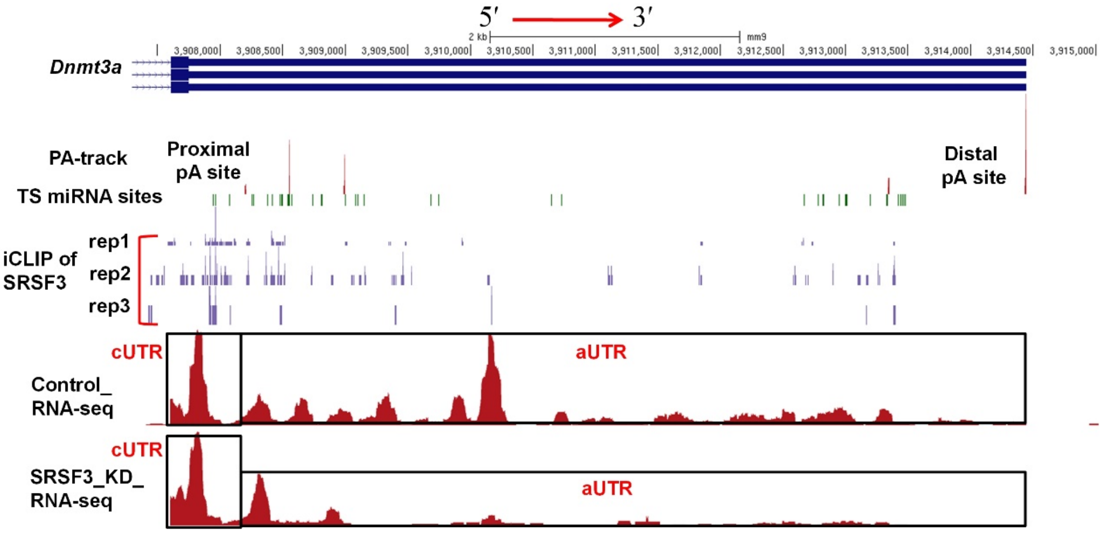

Supplementary Figure S8. SRSF3 preferentially binds around proximal pA site of *Dnmt3a* in mouse cells. A combination of PA-seq track, TargetScan predicted microRNA binding sties (TS miRNA sites), three replicates (rep1, rep2 and rep3) of SRSF3 iCLIP track, RNA-seq track of control (Ctrl) and *SRSF3*-KD cells near the 3′ UTR of mouse *Dnmt3a*. cUTR and aUTR represent common and alternative 3′ UTR of *Dnmt3a*, respectively. Red arrow at the top represents the transcription direction.

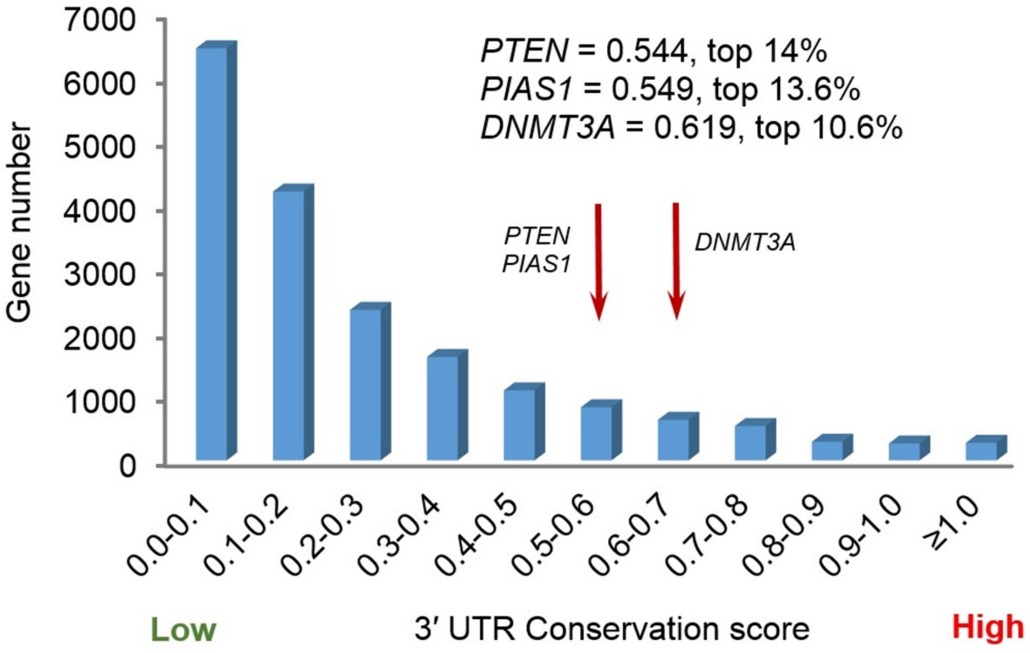

Supplementary Figure S9. Higher 3′ UTR conservation score for three candidate genes. The conservation score of 3′ UTR is based on the alignment of human genes to other vertebrates, and the supporting data are downloaded from Vertebrate Multiz Alignment & Conservation (100 Species) in USCS genome browser.

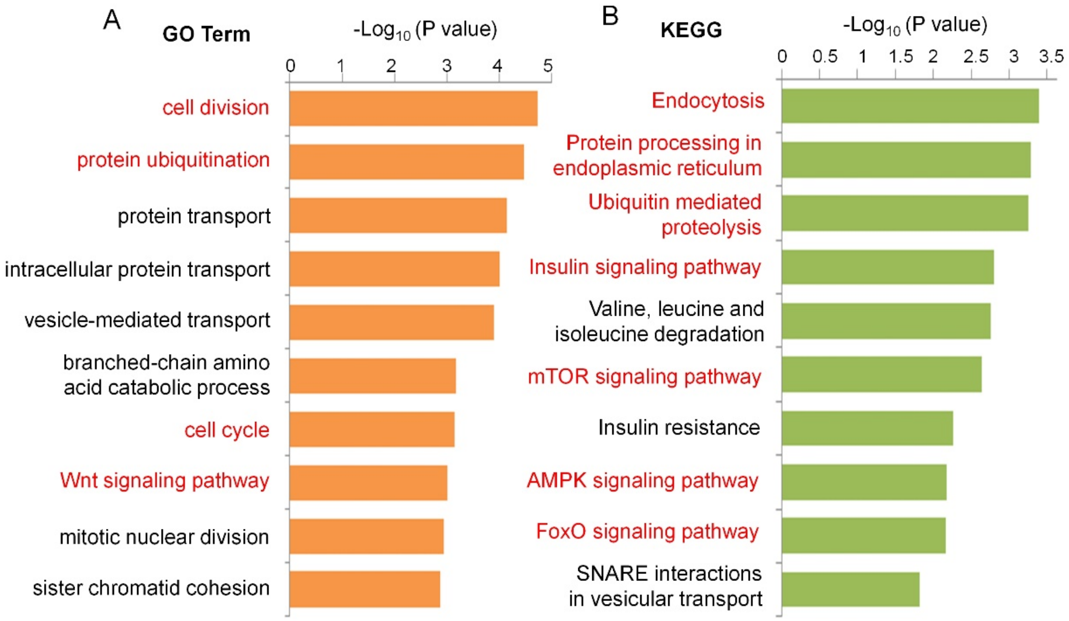

Supplementary Figure S10. GO (A) and KEGG (B) enrichment analyses for shared genes with shortened 3′ UTR in two SRSF3-knockdown replicates of HUVECs. Genes with ΔRUD ≤ -0.05 were used for analysis. Red fonts represent the GO terms and pathways related to senescence or aging.

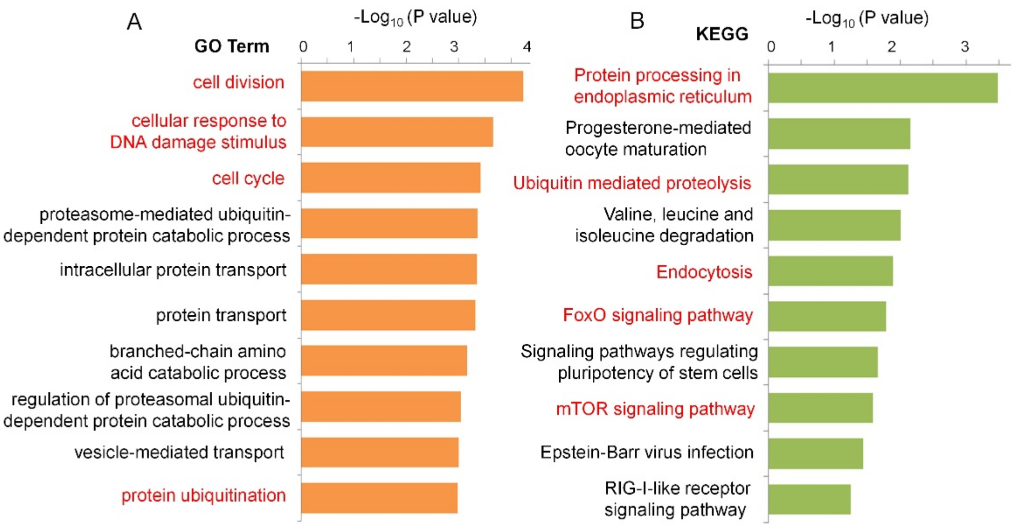

Supplementary Figure S11. GO (A) and KEGG (B) enrichment analyses for shared genes with shortened 3' UTR in SRSF3-KD 293T and HUVEC cells. Genes with ΔRUD ≤ -0.05 were used for analysis. Red fonts represent the GO terms and pathways related to senescence or aging.

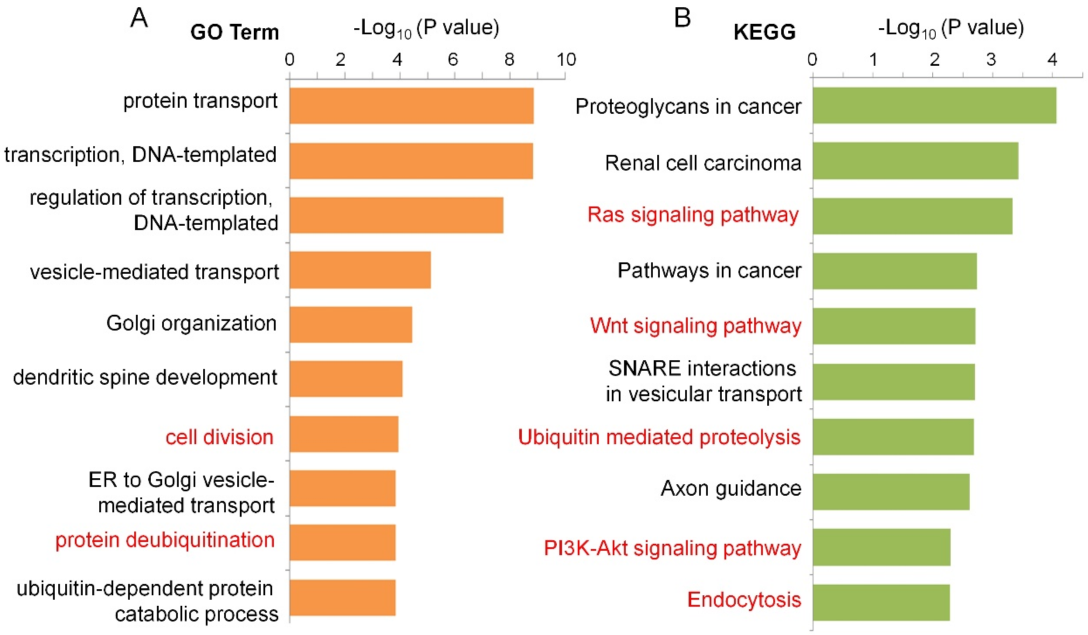

Supplementary Figure S12. GO (A) and KEGG (B) enrichment analyses for genes with shortened 3' UTR in MEFs. Genes with ΔRUD ≤ -0.1 were used for analysis. Red fonts represent the GO terms and pathways related to senescence or aging.

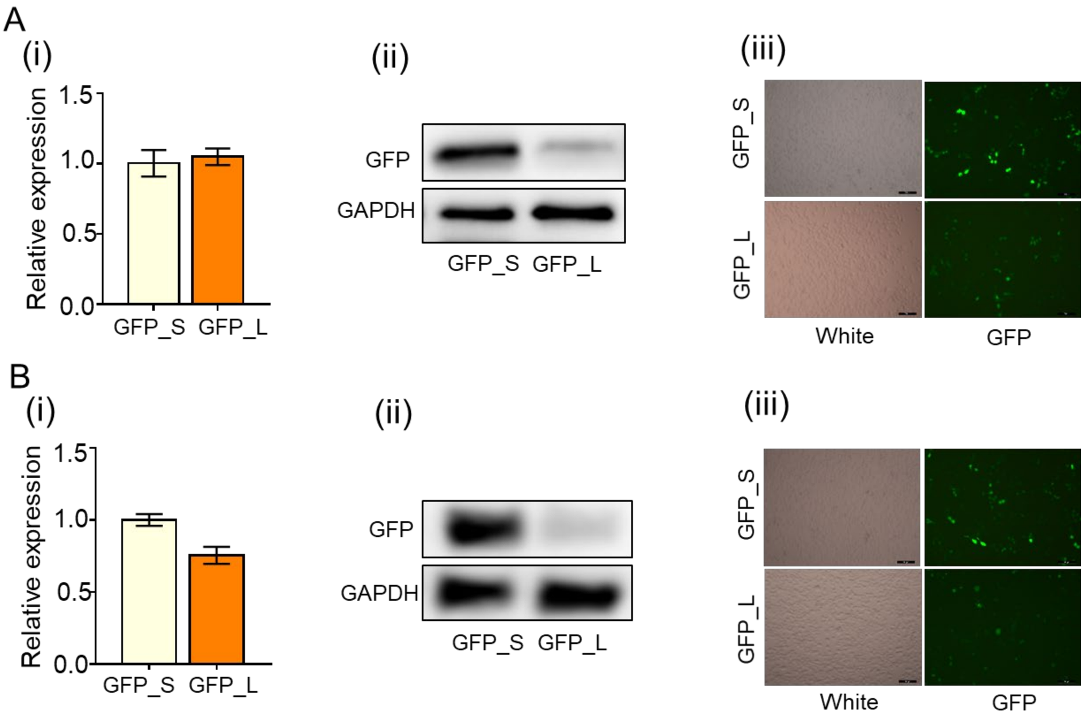

Supplementary Figure S13. GFP tagged with short 3′ UTR of *PTEN* produced more protein. (*A-B*) The detection of GFP RNA, protein and fluorescence intensity by qPCR (**i**), Western blot (**ii**) and fluorescence microscope (**iii**) for GFP_S and GFP_L in 293T cells. GAPDH served as internal control of Western blot. A and B represent two biological replicates respectively. GFP_S and GFP_L mean GFP coding sequence tagged with short and long 3′ UTR of *PTEN*, respectively.

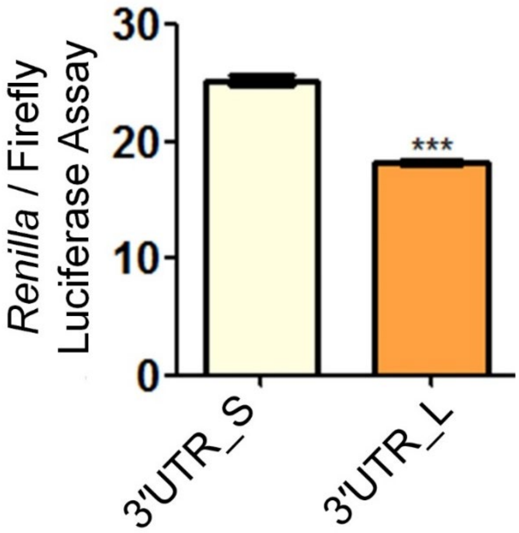

Supplementary Figure S14. Dual luciferase assay indicates shorter (S) 3′ UTR of *Pten* produces more protein than longer one (L) in mouse NIH3T3 cells.

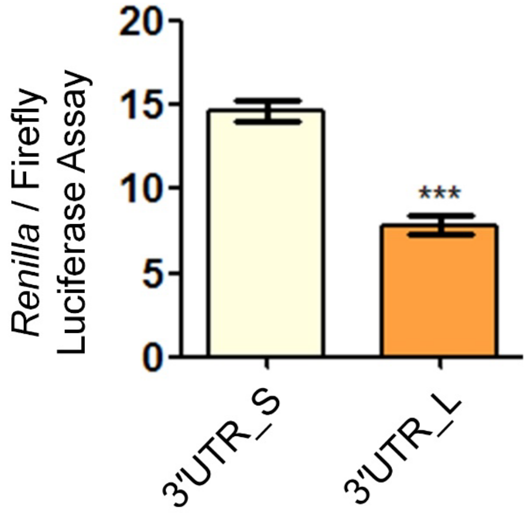

Supplementary Figure S15. Dual luciferase assay indicates shorter (S) 3' UTR of *Pias1* produces more protein than longer one (L) in mouse NIH3T3 cells.

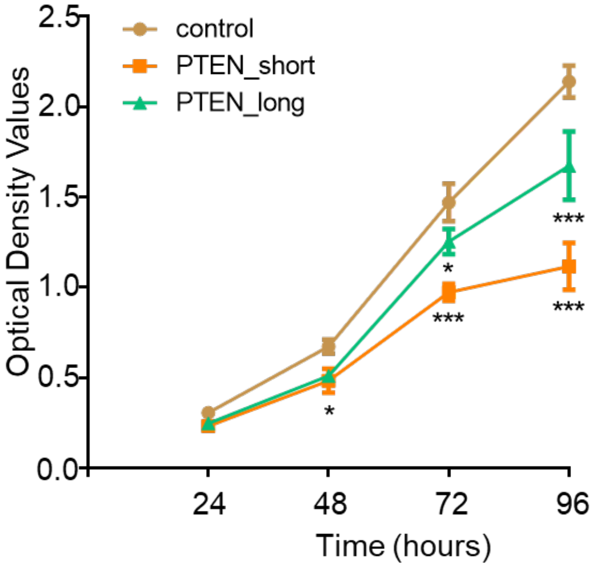

Supplementary Figure S16. Overexpression of *PTEN* with shorter 3'UTR has much stronger effect in reducing cell growth rate than the longer one. CCK-8 assay to evaluate cell proliferation after transfecting short, long isoform of *PTEN* and control vector to 293T cells. OD values were quantified after transfecting 24, 48, 72 and 96 hours, respectively. * and *** represent p value (*t*-test) less than 0.05 and 0.001, respectively.

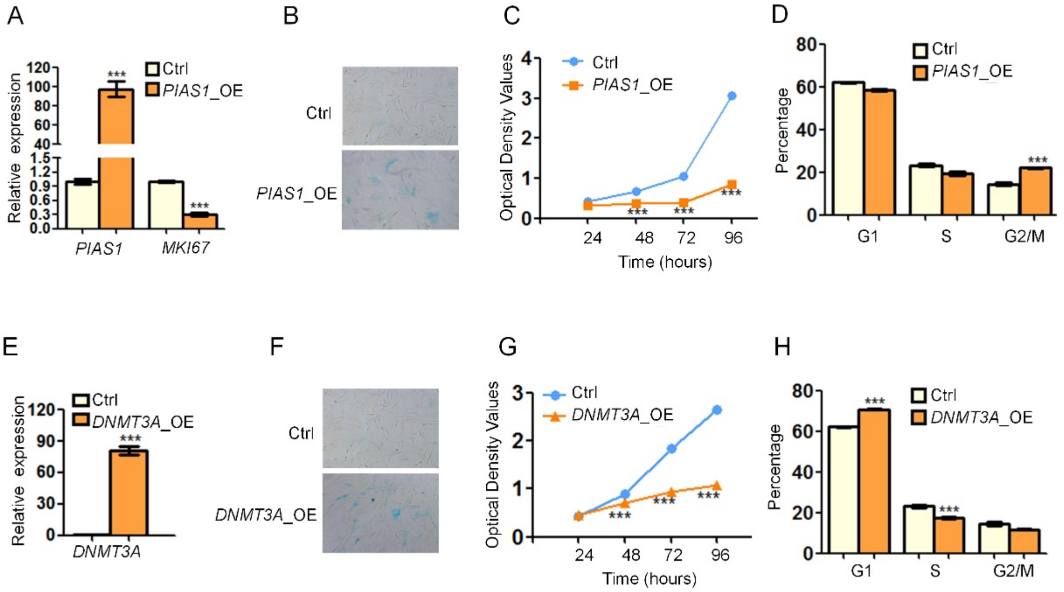

Supplementary Figure S17. Upregulation of other two APA-regulated genes (*PIAS1* and *DNMT3A*) contributes to senescence-associated phenotypes. (**A, E**) qRT-PCR indicated overexpression of *PIAS1* or *DNMT3A* (*PIAS1*_OE in A and *DNMT3A*_OE in E) led to decreased expression of cell proliferation marker *MKI67* in mouse cells. (**B, F**) SA-β-Gal staining of mouse cells overexpressing *PIAS1* (B) or *DNMT3A* (F). (**C, G**) CCK-8 assay for cells with or without overexpression (OE) of *PIAS1* (C) and *DNMT3A* (G) in mouse cells. (**D, H**) Cell cycle analysis before and after overexpression of *PIAS1* (D) and *DNMT3A* (H) in mouse cells. *** means P value less than 0.001 (*t*-test).

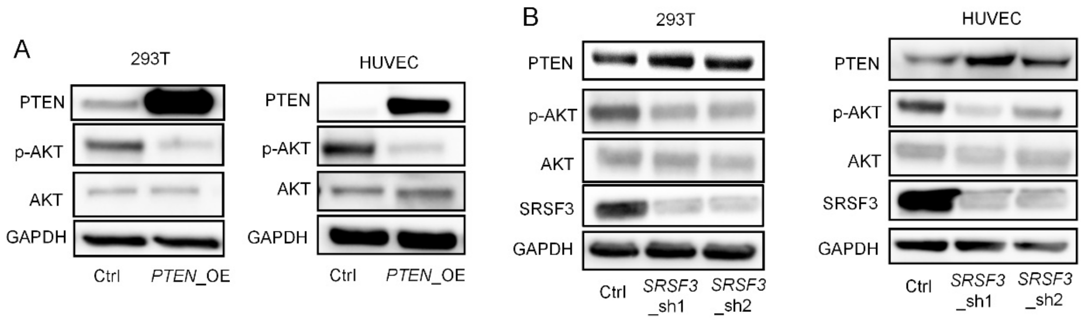

Supplementary Figure S18. *SRSF3*-KD induced upregulation of PTEN reduces phosphorylated form of AKT. (**A**) Western blot showed that overexpression of *PTEN* (*PTEN*_OE) reduced p-AKT level without obvious change in AKT protein abundance in 293T and HUVEC cells. (**B**) Western blot demonstrated that knockdown of *SRSF3* by two shRNAs (sh1 and sh2) significantly increased PTEN protein level and reduced p-AKT protein level in both human 293T and HUVEC cells. GAPDH served as internal loading control.

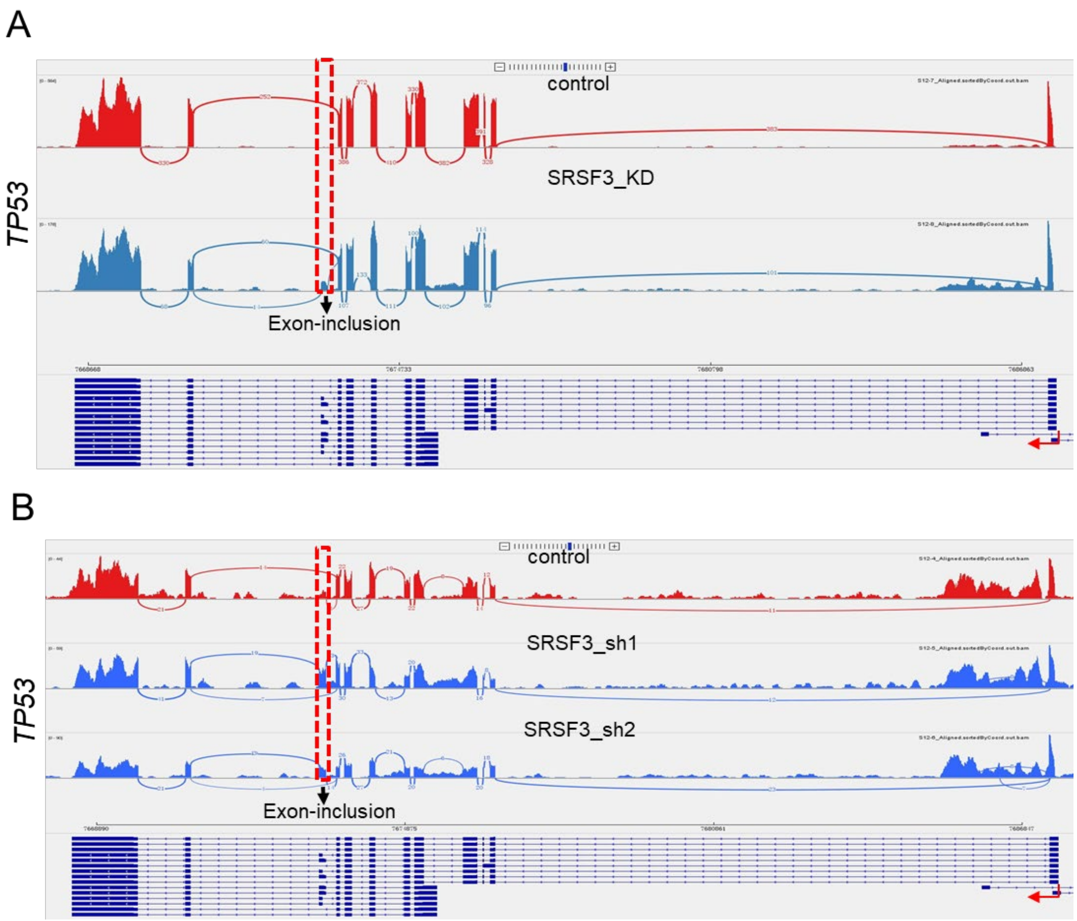

Supplementary Figure S19. The splicing pattern of *TP53* in *SRSF3*-knockdown and the control cells. Splicing pattern of *TP53* in 293T (A) and HUVEC (B). The dashed red rectangle represents that exon inclusion occurred in *TP53* to induce increased expression of the beta isoform of *TP53* in the SRSF3-KD. The curves between two exons indicate splicing junction. The red arrow indicates the transcription direction.

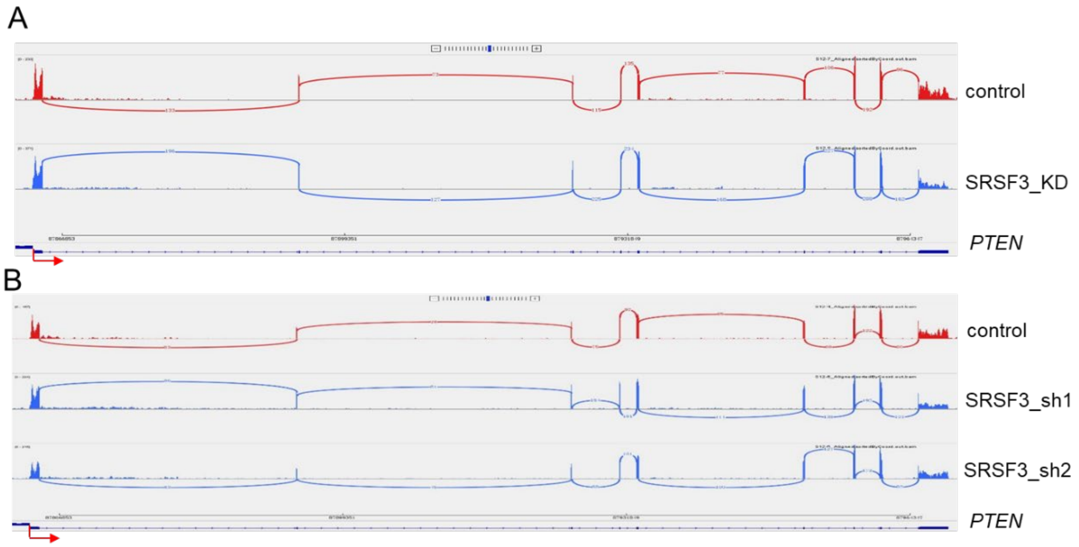

Supplementary Figure S20. RNA-seq splicing track of *PTEN* before and after knockdown of *SRSF3* in human 293T (**A**) and HUVEC cells (**B**). The curves between two exons indicate splicing junction. The red arrow indicates the transcription direction.

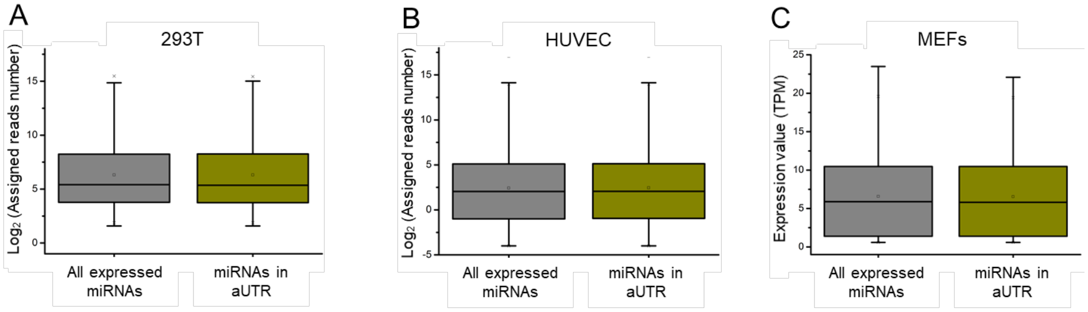

Supplementary Figure S21. Box plot for expression profiles of all miRNAs and aUTR-targeting miRNAs in human (293T and HUVEC) and mouse (MEFs) cells. (**A-B**) miRNA expression data in human cells, where log_2 transformed assigned reads numbers were used to reflect miRNA expression (293T in panel (A) and HUVEC in panel (B)). (**C**) miRNA expression data in MEFs, where TPM (transcript per million reads) was used as miRNA expression. aUTR means alternative 3′ UTR. miRNAs targeting aUTRs were predicted based on published methods [2, 3]. The miRNA expression data were downloaded from NCBI GEO database under accession number of GSE105414, GSE94410 and GSE52950 for 293T, HUVEC and MEFs, respectively.

Supplementary References

1. Hu J, Lutz CS, Wilusz J, Tian B. Bioinformatic identification of candidate cis-regulatory elements involved in human mRNA polyadenylation. RNA. 2005; 11:1485–93. https://doi.org/10.1261/rna.2107305

2. Bartel DP. MicroRNAs: target recognition and regulatory functions. Cell. 2009; 136:215–33. https://doi.org/10.1016/j.cell.2009.01.002

3. Sandberg R, Neilson JR, Sarma A, Sharp PA, Burge CB. Proliferating cells express mRNAs with shortened 3′ untranslated regions and fewer microRNA target sites. Science. 2008; 320:1643–47. https://doi.org/10.1126/science.1155390
