# Supplementary material for: Alternative polyadenylation dependent function of splicing factor SRSF3 contributes to cellular senescence
Source: Aging (Albany NY). 2019 Mar 4;11(5):1356–88. doi: 10.18632/aging.101836 (PMC6428108; doi:10.18632/aging.101836)
Supplement: Supplementary Table [file aging-11-101836-s002.pdf]

Supplementary Table

Supplementary Table S1. Primers used for qRT-PCR, luciferase, overexpression and knockdown.

Primer Name	Primer Sequence (5´ to 3´)	Species	Type
H_GAPDH-F	ATGTTCGTCATGGGTGTGAA	human	qPCR
H_GAPDH-R	GTTGTCATGGATGACCTTGG	human	qPCR
H_SRSF3-F	CGGCTTTGCTTTTGTTGAAT	human	qPCR
H_SRSF3-R	TGGGCCACGATTTCTACTTC	human	qPCR
H-CDK1-F	CTTTTCCATGGGGATTCAGA	human	qPCR
H-CDK1-R	AGGCTTCCTGGTTTCCATTT	human	qPCR
H_Mki67-F	TCAAGACCCCAGTGAAGGAG	human	qPCR
H_Mki67-R	AGATGGCTGTTTTGCTGCAT	human	qPCR
H_CDKN1B-F	CATTTGGTGGACCCAAAGAC	human	qPCR
H_CDKN1B-R	TTCTGAGGCCAGGCTTCTT	human	qPCR
H_CDKN1A-F	CGACTGTGATGCGCTAATGG	human	qPCR
H_CDKN1A-R	CTGCCTCCTCCCAACTCATC	human	qPCR
M_GAPDH-F	AGGTCGGTGTGAACGGATTTG	mouse	qPCR
M_GAPDH-R	TGTAGACCATGTAGTTGAGGTCA	mouse	qPCR
M_Mki67-Forward	ACAGGCTCCGTACTTTCCAA	mouse	qPCR
M_Mki67-Reverse	ATCAGACTGCTGCTTTGCTG	mouse	qPCR
M_CDK1-qForward	GGGAATTGTGTTTTGCCACT	mouse	qPCR
M_CDK1-qReverse	TCGGTACCACAGCGTCACTA	mouse	qPCR
M_CDKN2D-F	GCTCTGGCTTTCGTGAACAT	mouse	qPCR
M_CDKN2D-R	GGAGAAGGTAGTGGGGTCCT	mouse	qPCR
M_CDKN1A-F	CGGTGGAACTTTGACTTCGT	mouse	qPCR
M_CDKN1A-R	CAGGGCAGAGGAAGTACTGG	mouse	qPCR
M_Lmnb1-F	AGGCGAAGAAGAGCGGTTAA	mouse	qPCR
M_Lmnb1-R	AATGCTAACACTGCTGCTCG	mouse	qPCR
M_SRSF-F	TGATTACCGCAGGAGGAGTC	mouse	qPCR
M_SRSF3-R	GATCGAGACGGCTTGTGATT	mouse	qPCR
M_CCNB1-F	GGCTGACCCAAACCTCTGTA	mouse	qPCR
M_CCNB1-R	GGTCTCCTGAAGCAGCCTAA	mouse	qPCR
H-PTENshort-F	GCGTGCAGATAATGACAAGG	human	qPCR
H-PTENshort-R	GCTAGCCTCTGGATTTGACG	human	qPCR
H-PTENlong-F	TTTGGATGTGCAGCAGCTTA	human	qPCR
H-PTENlong-R	ATTTGATGCTGCCGGTAAAC	human	qPCR
H_NCOA1short-F	CAATCTGCAGCCATTCTTCA	human	qPCR
H_NCOA1short-R	TGTCCAACTGTTCTGCCTTG	human	qPCR
H_NCOA1long-F	ACAGTGGAAGAGGAGCATGG	human	qPCR
H_NCOA1long-R	AGCCCTTCCACCAATCTTTT	human	qPCR
H_Rbm25short-F	TGTTGCCATGGTACTTGATGA	human	qPCR
H_Rbm25short-R	CCTTAAAAGGGTGGCAAAGA	human	qPCR
H_Rbm25long-F	TTGGTGGGGTCCTTAAAACA	human	qPCR
H_Rbm25long-R	GAGAAGCTGAAGCAGCCATC	human	qPCR
H_DHX36short-F	ACACAGGAAAAGGCAACTCC	human	qPCR
H_DHX36short-R	TTGTTCATGTCCCAGGGTTT	human	qPCR
H_DHX36long-F	TTCTGTGCAGCTCTGTGTCC	human	qPCR
H_DHX36long-R	CACTTCTCAGCTGCCATTCA	human	qPCR
H_LAMC1short-F	GGACATTCGCAATCTGGAGG	human	qPCR
H_LAMC1short-R	TGCCTTCCAGCCCTAAAGAC	human	qPCR
H_LAMC1long-F	CCGCAACACAGCCATTATTT	human	qPCR
H_LAMC1long-R	ATCCCTGTGTCAACCAGCAT	human	qPCR
H_PCGF5short-F	CCTTTTGCCAAGAATTCCAA	human	qPCR
H_PCGF5short-R	TGTGATCAGCTGCCAACATT	human	qPCR
H_PCGF5long-F	AATGGGTCACAGAACAAGCA	human	qPCR

H_PCGF5long-R	TGCATCCATTTTCACCCTATC	human	qPCR
H_CUL4Bshort-F	CGGTTGAAGAACAAGCAAGC	human	qPCR
H_CUL4Bshort-R	TCCATGTAGTCCCGGTCAAT	human	qPCR
H_CUL4Blong-F	TGTTGTTGAACTCTTTGCATGTT	human	qPCR
H_CUL4Blong-R	AAGCAAATAAACCTTTTGCTTCA	human	qPCR
H_IGF2BP1short-F	AAAAACGGTGAACGAGTTGC	human	qPCR
H_IGF2BP1short-R	CCAGGATGTCTCGGATCTTC	human	qPCR
H_IGF2BP1long-F	ACATATGGAAAAGCCCATGC	human	qPCR
H_IGF2BP1long-R	GGGGGAACAAAAAGGAAAAT	human	qPCR
H_DDX5short-F	TCCAACAGGGACTTACCAGAA	human	qPCR
H_DDX5short-R	GAATATCCTGTTGGCATTGGA	human	qPCR
H_DDX5long-F	TGTGAGAACGTGGGTGACTT	human	qPCR
H_DDX5long-R	AGCTTAGCTCCTGGCCATCT	human	qPCR
H_HOXC9short-F	AGACGCTGGAACTGGAGAAG	human	qPCR
H_HOXC9short-R	AGGCTGGGTAGGGTTTAGGA	human	qPCR
H HOXC9long-F	GCTCTGCGTGCAGATTTTGT	human	qPCR
H_HOXC9long-R	GGGGATGAGAGGGAACACTA	human	qPCR
H_RAB2Ashort-F	ATTAAAATTGGCCCTCAGCA	human	qPCR
H_RAB2Ashort-R	GAGGAGAGGGGGTGAAAGAA	human	qPCR
H_RAB2Along-F	GGAATTGCTTGGACACTGAA	human	qPCR
H_RAB2Along-R	GGCAAACAAATCCCTTTCTG	human	qPCR
M_DNMT3Ashort-F	TCTCAACAGCACCATTCCTG	mouse	qPCR
M_DNMT3Ashort-R	TGTGTGGTAGGCACCTGAAA	mouse	qPCR
M_DNMT3Along-F	TAGAAGGGCCGTCTGTATGC	mouse	qPCR
M_DNMT3Along-R	CTTTCCCAGTCTGCTCAAGG	mouse	qPCR
M_CREB1short-F	AAGCAGCACGGAAGAGAGAG	mouse	qPCR
M_CREB1short-R	TTTCAAGCACTGCCACTCTG	mouse	qPCR
M_CREB1short-F	CCCCAGTGGAACCTAGACAT	mouse	qPCR
M_CREB1short-R	CCCTGTTTTGTGCTGACAGT	mouse	qPCR
M_PIAS1short-F	TCCCTGTCTCCTACGTCACC	mouse	qPCR
M_PIAS1short-R	TAAGGCATAGGCGTCATGTG	mouse	qPCR
M_PIAS1long-F	GGATGGGTTTGGGATAGCTT	mouse	qPCR
M_PIAS1long-R	ACAGGTACACAGGCGCTCTT	mouse	qPCR
M_FOSL1short-F	GGAACTGCACTGGATGGTG	mouse	qPCR
M_FOSL1short-R	CCCTAGGGCTCGTATGACTC	mouse	qPCR
M_FOSL1long-F	GGCAGCCCAGACTTTGTAGA	mouse	qPCR
M_FOSL1long-R	TCAAAGGGCACCTTCTGTCT	mouse	qPCR
M_HDAC4short-F	CGCTATGACGATGGGAACTT	mouse	qPCR
M_HDAC4short-R	CATCTGGGGCAAACTCATTT	mouse	qPCR
M_HDAC4long-F	AGATGCCATTTCCCAATCTG	mouse	qPCR
M_HDAC4long-R	TGGAGCACACACAGCTTCAT	mouse	qPCR
M_E2F3short-F	ATTTGGAAAAGCTGCCTCTG	mouse	qPCR
M_E2F3short-R	GGACAACACTGCGATACACG	mouse	qPCR
M_E2F3long-F	TTCTGTTGTTTTCGCCTGTG	mouse	qPCR
M_E2F3long-R	TGCACACGCTTGGATTCTTA	mouse	qPCR
M_PTENshort-F	TGGCAATAGGACATTGTGTCA	mouse	qPCR
M_PTENshort-R	ACAAGTGTCAAAACCCTGTGG	mouse	qPCR
M_PTENlong-F	TCGGGAATTTGGTGTCTTTC	mouse	qPCR
M_PTENlong-R	GATGGCGTTCTGCCTAATCT	mouse	qPCR
M_MECP2short-F	AACAGAGAGGAGCCTGTGGA	mouse	qPCR
M_MECP2short-R	AATCGGGAAGCTTTGTCAGA	mouse	qPCR
M_MECP2long-F	CAGGCTGAGGAGACATCACA	mouse	qPCR
M_MECP2long-R	CCTAGCCCCCTTCAGTTTTT	mouse	qPCR
M_TRAF3short-F	CTGACCCCTGACAAGAAAGC	mouse	qPCR
M_TRAF3short-R	CTCGACCTCCTTCTCCTCCT	mouse	qPCR
M_TRAF3long-F	CCCTCTCAGCTCACTGTTCC	mouse	qPCR

M_TRAF3long-R	TGGTTTGCACACATTGGTTT	mouse	qPCR
M_SSBP3short-F	TGCAAGAAGTGAGGTGTCCA	mouse	qPCR
M_SSBP3short-R	GAGAGTTTTCTGGCCGTTGA	mouse	qPCR
M_SSBP3long-F	GATGGACAGACTTCGGCATT	mouse	qPCR
M_SSBP3long-R	GACAGGCTCCATCATCAGGT	mouse	qPCR
M_NEO1short-F	TGAACCAGATGAGCTGACCA	mouse	qPCR
M_NEO1short-R	AGGCTTGGAGTCATGTCCAG	mouse	qPCR
M_NEO1long-F	TGGGTGATTTTACCCCTTTG	mouse	qPCR
M_NEO1long-R	CCGTCATGTGACCAGCTAGA	mouse	qPCR
M_RASA2short-F	AAGAATGGACATCGGCTTTG	mouse	qPCR
M_RASA2short-R	TTCCCATCAGAAGGAAGCTG	mouse	qPCR
M_RASA2long-F	CAGAGTCCAAGGACAGTCAGG	mouse	qPCR
M_RASA2long-R	TTGCAGAAGCAGAGAAAGGAG	mouse	qPCR
M_RAB2Ashort-F	CCAAGAAGGGGTCTTTGACA	mouse	qPCR
M_RAB2Ashort-R	AGCTAGCCGGTAACACCAGA	mouse	qPCR
M_RAB2Along-F	GAGACCGCTTCACAAAAGGA	mouse	qPCR
M_RAB2Along-R	GGGAAGAGTAAGTGGCATGG	mouse	qPCR
M_DHX36short-F	CCCTGGGTTATGACTTGCTT	mouse	qPCR
M_DHX36short-R	TGGGAATTCTCCCAAAGTGT	mouse	qPCR
M_DHX36long-F	TGCTGGTTCAGGTACTGCTG	mouse	qPCR
M_DHX36long-R	CCTTGACTTTAGGGGCAAAA	mouse	Luciferase
M_PTENshortUTR-F	CCGCTCGAGCCAGAGAATGAACCTT TTGATG	mouse	Luciferase
M_PTENshortUTR-R	CGGGTTTAAACTGGAATAAAATGGG AAAGTGC	mouse	Luciferase
M_PTENlongUTR-F	CGGGTTTAAACTGACAAGAATGAGA CTTTAATC	mouse	Luciferase
M_PIAS1shortUTR-F	CCGCTCGAGCGCTCTCACCCTGCTG CT	mouse	Luciferase
M_PIAS1shortUTR-R	CGGGTTTAAACTGAAAATATAGTTTT GTTCTC	mouse	Luciferase
M_PIAS1longUTR-R	CGGGTTTAAACAACTGATAACAAAT GTCTTG	mouse	Luciferase
H_PTENshortUTR-F	CCGCTCGAGTAGAGGAGCCGTCAAA TCCA	human	Luciferase
H_PTENshortUTR-R	CGGGTTTAAACTCAGTTTATTCAAGT TTATT	human	Luciferase
H_PTENlongUTR-R	CGGGTTTAAACTGACAAGAATGAGA CTTTAA	human	Luciferase
H_PTENOE-F	CGGAATTCGCCACC TTCCATCCTGCAGAAGAAGC	human	Over expression
H_PTENOE-R	CGCGGATCC CTTATCGTCGTCATCCTTGTAATC GACTTTTGTAATTTGTGTATGCTGA	human	Over expression
M_PTENOE-F	GAATTCGCCACCACAGGCTCCCAGA CATGACA	mouse	Over expression
M_PTENOE-R	GGATCCGACTTTTGTAATTTGTGAAT GCTGA	mouse	Over expression
M_PIAS1OE-F	GAATTCGCCACCCAAGATGGCGGAC AGTGC	mouse	Over expression
M_PIAS1OE-R	GGATCCGTCCAATGAGATAATGTCTG G	mouse	Over expression
M_DNMT3AOE-F	GAATTCGCCACCGCCTACTGCCCAG CAATG	mouse	Over expression
M_DNMT3AOE-R	GGATCCCACACAAGCAAAATATTCC TTCAGC	mouse	Over expression
H_SRSF3shRNA1-F	CCGGTGGAACTGTCGAATGGTGAAA	human	Knock down

	CTCGAGTTTCACCATTCGACAGTTCC ATTTTTG		
H_SRSF3shRNA1-R	AATTCAAAAATGGAACTGTCGAATG GTGAAACTCGAGTTTCACCATTCGA CAGTTCCA	human	Knock down
H_SRSF3shRNA2-F	CCGGCGAGAGCTAGATGGAAGAAC ACTCGAGTGTTCTTCCATCTAGCTCT CGTTTTTG	human	Knock down
H_SRSF3shRNA2-R	AATTCAAAAACGAGAGCTAGATGGA AGAACACTCGAGTGTTCTTCCATCTA GCTCTCG	human	Knock down
M_SRSF3shRNA1-F	CCGGTAAGAGTGGAACTGTCGAATG CTCGAGCATTCGACAGTTCCACTCTT ATTTTTG	mouse	Knock down
M_SRSF3shRNA1-R	AATTCAAAAATAAGAGTGGAACTGT CGAATG CTCGAGCATTCGACAGTTCCACTCTT A	mouse	Knock down
M_SRSF3shRNA2-F	CCGGGCGGAAATTGAAAGAAGCTAT CTCGAGATAGCTTCTTTCAATTTCCG CTTTTTG	mouse	Knock down
M_SRSF3shRNA2-R	AATTCAAAAAGCGGAAATTGAAAGA AGCTATCTCGAGATAGCTTCTTTCAA TTTCCGC	mouse	Knock down
